# Mass Spectrometry Imaging Reveals Early Metabolic Priming of Cell Lineage in Differentiating Human-Induced Pluripotent Stem Cells

**DOI:** 10.1021/acs.analchem.2c04416

**Published:** 2023-03-10

**Authors:** Arina A. Nikitina, Alexandria Van Grouw, Tanya Roysam, Danning Huang, Facundo M. Fernández, Melissa L. Kemp

**Affiliations:** † School of Biological Sciences, Georgia Institute of Technology, Atlanta, Georgia 30332, United States; ‡ School of Chemistry and Biochemistry, Georgia Institute of Technology, Atlanta, Georgia 30332, United States; § The Wallace H. Coulter Department of Biomedical Engineering, Georgia Institute of Technology and Emory University, Atlanta, Georgia 30332, United States; ∥ Petit Institute of Bioengineering and Biosciences, Georgia Institute of Technology, Atlanta, Georgia 30332, United States

## Abstract

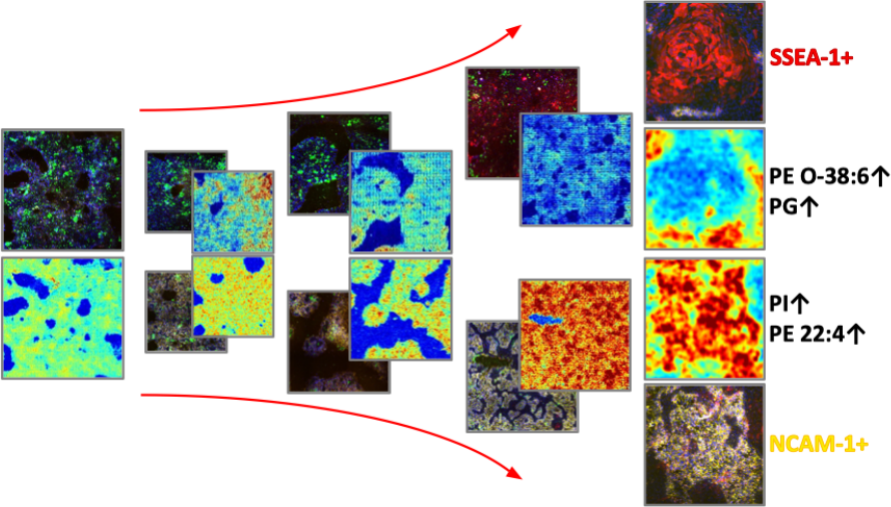

Induced pluripotent stem cells (iPSCs) hold great promise in regenerative medicine; however, few algorithms of quality control at the earliest stages of differentiation have been established. Despite lipids having known functions in cell signaling, their role in pluripotency maintenance and lineage specification is underexplored. We investigated the changes in iPSC lipid profiles during the initial loss of pluripotency over the course of spontaneous differentiation using the co-registration of confocal microscopy and matrix-assisted laser desorption/ionization (MALDI) mass spectrometry imaging. We identified phosphatidylethanolamine (PE) and phosphatidylinositol (PI) species that are highly informative of the temporal stage of differentiation and can reveal iPS cell lineage bifurcation occurring metabolically. Several PI species emerged from the machine learning analysis of MS data as the early metabolic markers of pluripotency loss, preceding changes in the pluripotency transcription factor Oct4. The manipulation of phospholipids via PI 3-kinase inhibition during differentiation manifested in the spatial reorganization of the iPS cell colony and elevated expression of NCAM-1. In addition, the continuous inhibition of phosphatidylethanolamine *N*-methyltransferase during differentiation resulted in the enhanced maintenance of pluripotency. Our machine learning analysis highlights the predictive power of lipidomic metrics for evaluating the early lineage specification in the initial stages of spontaneous iPSC differentiation.

## Introduction

Induced pluripotent stem cells (iPSCs) can be reprogrammed from a patient’s own adult cells^1^ and differentiated into any cell type with many potential clinical uses.^2−5^ Numerous in vitro studies have developed directed differentiation protocols, resulting in tissues of interest.^6−9^ In contrast, spontaneous, or undirected, differentiation allows the production of all three germ lineages and can be used as a model of initial loss of pluripotency that is applicable to a wide range of protocols. Human iPSC colonies are disordered, unlike embryos, yet take on a degree of self-assembly and organization over time; however, the mechanisms of cellular reprogramming and colony self-organization are still understudied.

Quality control is a process that helps maintain safety, potency, and identity of cells during manufacturing. When iPSCs are used for regenerative medicine therapies, quality control and a thorough understanding of the mechanisms responsible for cell fate decisions are essential to prevent teratomas, reduce heterogeneity in the differentiated phenotypes, and accelerate timelines for maturation protocols.^10^ In a cell manufacturing setting, typical quality control includes the initial confirmation of cellular pluripotency by confirming sufficient Oct4 expression in the colony sample.^11^ After a differentiation protocol is completed, quality control can include quantifying the expression levels of phenotype marker genes by flow cytometry as well as tissue functional tests (e.g., contractility in cardiomyocytes, production of collagen in fibroblasts, etc.). A more extensive quality control of the finalized clinical treatment can include whole genome sequencing and whole exome sequencing.^5^ Endpoint assays confirm the cellular state prior to patient delivery, yet robust strategies to evaluate the early loss of differentiation are needed in cell manufacturing industry applications.

Most of the described approaches are destructive, with only several known glycoprotein surface markers allowing real-time quality control.^12^ To date, quality control has rarely been performed by assessing cellular lipids. Recently, the expression of plasmalogens and sphingomyelins was shown to increase during the process of iPSC differentiation into vascular endothelial cells,^13^ suggesting that phospholipid metabolism plays an important role. In addition to their well-known contribution to the structure in membranes, polyunsaturated phospholipids are precursors of critical signaling molecules,^14^ and lipid supplementation was previously shown to influence the general iPSC phenotype.^15^ Here, we focus on phosphatidic acids (PA) and glycerophospholipids such as phosphatidylethanolamines (PE), phosphatidylcholines (PC), phosphatidylserines (PS), and phosphatidylinositols (PI). In addition to functioning as the negatively charged building blocks of membranes, phosphatidylinositols and related phosphates facilitate interfacial binding of proteins and regulate protein activity at the cell interface. A well-known example is the Akt/PKB signaling pathway, which is activated by the PI 3-kinase phosphorylation of phosphatidylinositols, followed by the recruitment of Akt to the membrane due to the interaction with the resulting phosphoinositide docking sites. Activated Akt then controls many key cellular functions, including differentiation, proliferation, metabolism, and apoptosis.

In this work, we assess the changes in phospholipid abundances in iPSCs over the course of the spontaneous differentiation protocol as well as their spatial distribution inside a colony using both high and ultrahigh resolution matrix-assisted laser desorption/ionization (MALDI) mass spectrometry (MS) imaging co-registered with confocal microscopy. MALDI MS imaging has been successfully used before to show that the distribution of phosphatidylcholines differs between the differentiated and undifferentiated parts of iPSC colonies.^16^ We developed a suite of machine learning models that indicate dynamic and spatial trends at the single-cell lipidome level and robustly predict pluripotency loss earlier than typical markers such as Oct4; furthermore, the lipidomic signatures capture bifurcation in lineage specification between SSEA1+ and NCAM1+ phenotypes.

## Experimental Section

### Co-Registration Sample Preparation

SYLGARD silicone (10:1 ratio with the curing agent) was poured in a custom-made 3D-printed molds and placed in a 70 °C oven for 3 h. The resulting eight-well silicone wall was adhered to an indium tin oxide (ITO)-coated slide with SYLGARD silicone, and the resulting culture slide (Figure S1) was placed in the oven for 30 min. HiPSCs were seeded as a monolayer at 2000 cells/mm^2^ density into a new Matrigel-coated well of the resulting eight-well slide every day to achieve staggered differentiation. To initiate spontaneous differentiation, the media was switched to RPMI plus B-27 supplement (49:1) the next day after seeding. For PI 3-kinase inhibition, LY294002 powder was reconstituted at 25 mM in DMSO and added to the RPMI/B-27 media at 35 or 100 μM during the first 24 h of spontaneous differentiation, after which the cells were fed fresh RPMI/B-27 media. For PEMT inhibition, 3-deazaadenozine powder was reconstituted at 50 mM in DMSO and added daily to fresh RPMI/B-27 media during feeds at 50 μM. The resulting samples had eight consecutive days of spontaneous differentiation on a single slide, with the shortest cell culture being pluripotent stem cells (0 days of differentiation) and the longest cell culture undergoing differentiation for 7 days. Next, the cells were incubated with Hoechst (1:1000), NL493-conjugated Mouse Anti-Human TRA-1-81, NL557-conjugated Mouse Anti-Human SSEA-1, and Alexa Fluor 647-conjugated Mouse Anti-Human NCAM-1/CD56 live stains diluted in media (1:50) for 30 min. Confocal images of live colonies were acquired on a Nikon UltraVIEW VoX W1 spinning disk confocal system with an sCMOS camera at 10x magnification (0.65 μm/px). Next, the cell culture media and silicone wall were removed, and samples were washed by submerging the plate into 5 mM ammonium formate buffer for 3 s to enhance spectral abundances. Norharmane was used as the MALDI matrix and deposited via sublimation. A slide containing cell colonies was taped to the bottom of the condenser in a simple sublimation apparatus. Solid norharmane was placed at the bottom of such a sublimation apparatus. Sublimation was performed at 250 °C under vacuum for 6 min. All experiments are summarized in Table S1.

### MALDI TOF MS Imaging

Matrix-deposited samples were analyzed in reflectron mode using a RapifleX Tissuetyper time of-flight (TOF) mass spectrometer (Bruker Daltonics, Billerica, MA, USA) equipped with a Smartbeam3D 10 kHz Nd:YAG (355 nm) laser. Imaging experiments were controlled by the FlexImaging 4.0 software (Bruker Daltonics, Billerica, MA, USA) using the single Smartbeam laser setting (∼5 μm in both x and y dimensions) with the laser raster size of 10 μm in both x and y dimensions. Data were collected in negative ion mode in the *m/z* 200–1600 range, with 200 laser shots averaged at each pixel. Mass calibration was performed using red phosphorus as a standard prior to data acquisition. Representative collected spectra are shown in Figure S8. Blank spectra are shown in Figure S9. All the detected features are listed in Table S3.

### MALDI FTICR MS Experiments

Ultrahigh mass resolution data were collected on a Bruker solariX 12-Tesla Fourier transform ion cyclotron resonance (FTICR) mass spectrometer equipped with a MALDI ion source. Data were acquired in negative mode from *m/z* 300 to 1200 at 1 M transient size with 25 μm raster width. The laser was set to minimum focus at 25% power. Real time calibration was employed with lock masses 333.11457 (deprotonated norharmane dimer) and 885.54986 (deprotonated PI 38:4). Data preprocessing was done in SCiLS Lab (SCiLS GmbH, Bremen, Germany) software. The mass spectra were preprocessed during import into SCiLS Lab by converting the spectra to centroid. MS/MS data were collected using quadrupole precursor mass selection. Collision energies ranged from 15 to 35 eV for selected peaks.

### Co-Registration

All MALDI MS data preprocessing was performed using the SCiLS Lab (SCiLS GmbH, Bremen, Germany) software. The mass spectra were preprocessed during import into SCiLS Lab using baseline removal by iterative convolution. A minimum interval width of 20 mDa around the average peak center was used to account for peak shifts throughout the experiment. Manual peak screening was performed to select the *m/z* features that were associated with the cell colony distribution. Next, we exploited and enhanced the multimodal image analysis approach^17^ previously developed in our lab to align the confocal and MALDI imaging data and extract cell-by-cell *m/z* spectra from imzML and .bd files generated by the RapifleX instrument. We used a confocal image stained with Hoechst nuclei live dye and a MALDI ion image averaged over the *m/z* spectrum as reference images for alignment. The algorithm rotates, shifts, and scales reference images in a given range of parameters until the global maximum of mutual information of the images is found. Confocal imaging was done at 0.65 μm/px, which allowed to extract and overlay nuclear outlines on scaled MALDI MS images (with the initial spatial resolution of 10 μm/px). As the size of an iPSC nucleus averages at 10 μm, this method approaches single-cell resolution.

## Results and Discussion

### Phospholipid Abundances Precede the Loss of Oct4 and Are Predictive of Metabolic Priming during Spontaneous Differentiation

**Figure 1:**
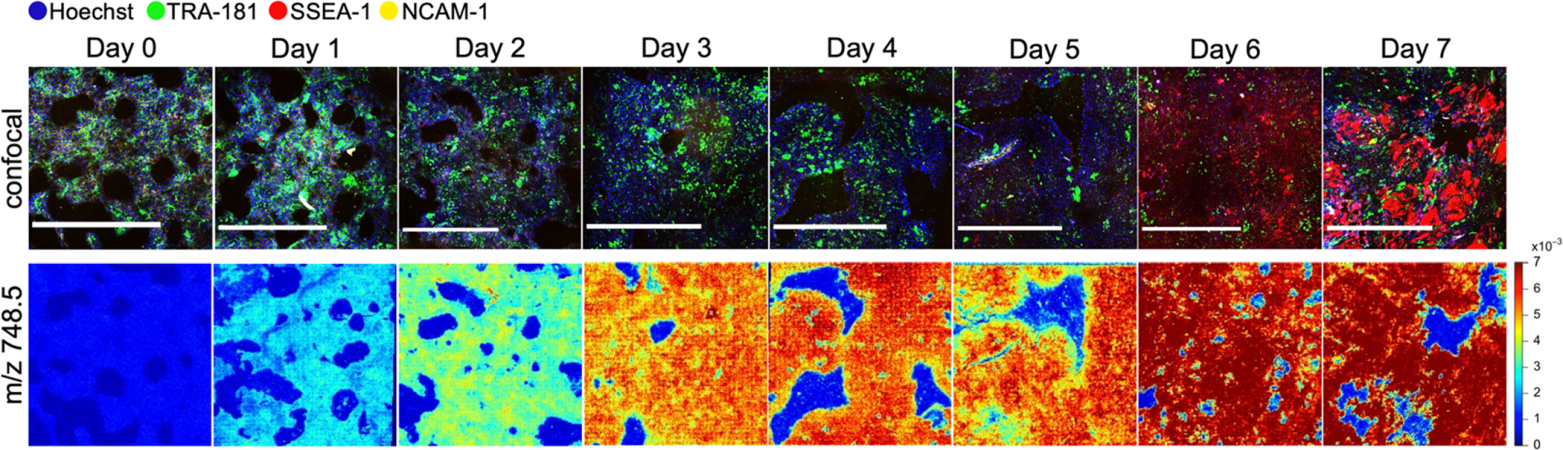
Co-registration of HiPSC colonies undergoing spontaneous differentiation. Top row––confocal images of iPSC colonies undergoing spontaneous differentiation for 7 days; bottom row––corresponding MALDI TOF ion images for *m/z* 748.5. Scale bar: 1 mm.

**Figure 2:**
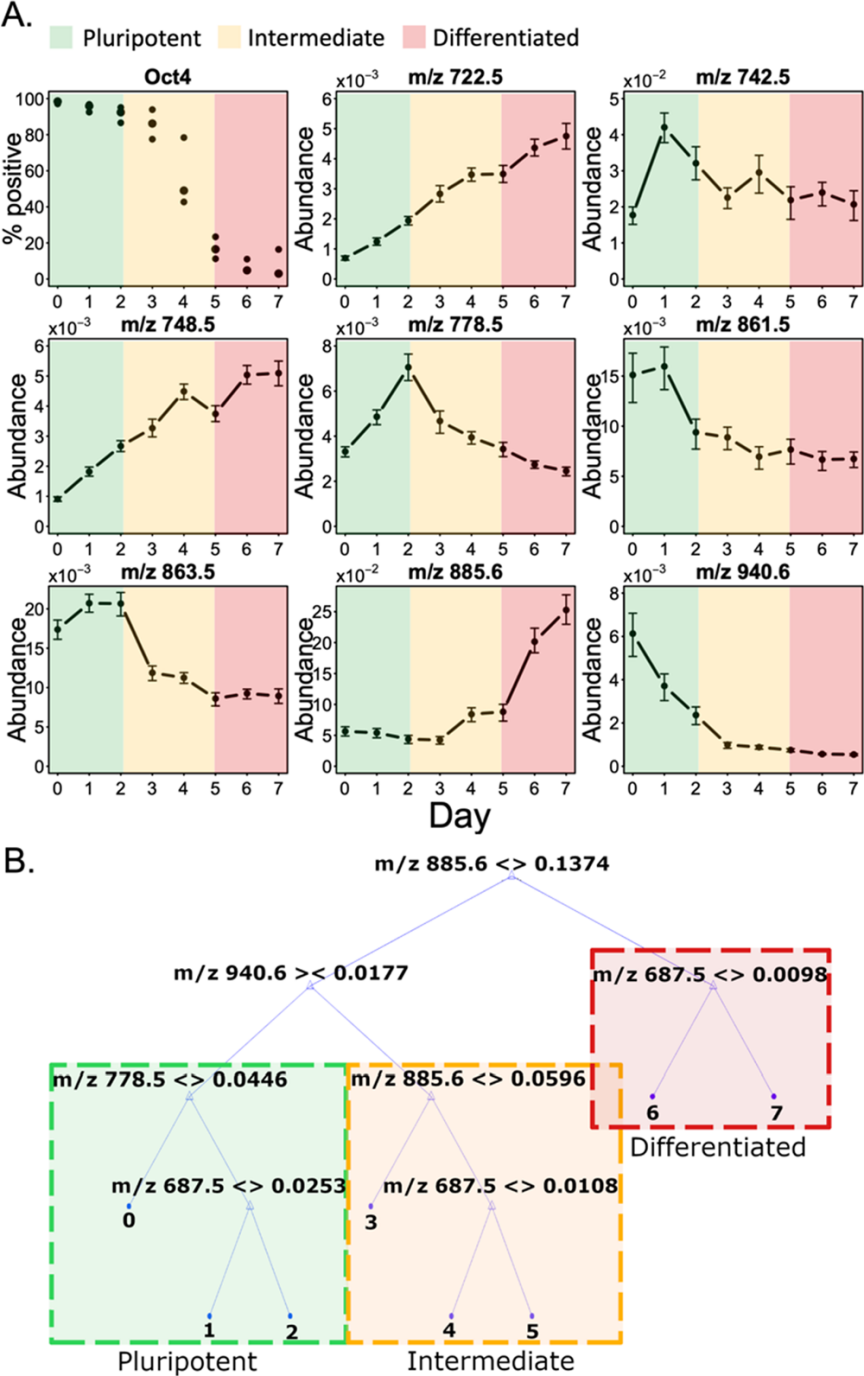
Degree of spontaneous differentiation of iPSC colonies can be predicted through a subset of metabolic features. (A) Temporal changes in Oct4-positive cells over 7 days of spontaneous differentiation measured by flow cytometry and eight examples of the corresponding changes in median phospholipid abundances. Percentage of Oct4-positive cells shown for three biological replicates; error bars in the phospholipid abundance plots show 25th and 75th percentiles. (B) Decision tree trained to predict the day of differentiation based on phospholipid abundance with the validation accuracy of 67% for classification into 8 days and 87% for classification into three major classes: pluripotent, undergoing differentiation, and differentiated.

To determine the dynamic changes in lipids during the loss of pluripotency in iPSCs, we analyzed iPSC colony samples undergoing 0–7 days of spontaneous differentiation protocol. For each of eight consecutive days of spontaneous differentiation, confocal microscopy and MS images of the same ROI were acquired and aligned (Figure 1). Next, the nuclei in each confocal image were segmented and their contours overlaid on the MS images: the average signal for each selected *m/z* value was then calculated for each nucleus. This protocol yielded eight datasets on the order of 10^4^ cells and 70 *m/z* peak-picked features each. To assess the temporal changes in phospholipid abundance occurring during the pluripotency loss, we calculated the average signal per day of differentiation for each of the *m/z* features. Eight representative trajectories of interest are shown in Figure 2a: the abundances of *m/z* 722.5 and 748.5 exhibited stable growth with the differentiation time, while the abundances of *m/z* 742.55, 778.53, 861.5, and 863.5 showed some initial growth but declined for the remaining differentiation times. The abundance of *m/z* 885.6 was stable for the first 4 days after which it exhibited rapid growth, making it anticorrelated (*R* = −0.85) with the pluripotent factor Oct4 expression levels measured via flow cytometry (Figure 2a, top left panel). The abundance of *m/z* 940.6 rapidly decreased to near-zero values in the first 4 days of differentiation, preceding the reduction in Oct4 expression, suggesting that this species could be used as an early metabolic marker of pluripotency loss. Partial least-squares regression (PLSR) of the differentiation day against phospholipid abundances yielded a validation *R*^2^ of 0.84. PLSR scores shown in the biplot in Figure S2 reveal distinct clusters for days 6 and 7, while days 4 and 5 cluster together, same as days 0 and 1. PLSR loadings shown in the same biplot form 2 distinct clusters corresponding to *m/z* values whose abundances increase versus *m/z* values whose abundances decrease with the differentiation time. Next, to identify critical *m/z* values that are the most predictive of the differentiation stage as well as to create a simple interpretable model, we trained a decision tree classifier (Figure 2b) using cell-by-cell lipid abundances as features and the day of differentiation as a class label. We used a biological replicate of the same experiment as a validation dataset, which yielded 67% validation accuracy when classified into 8 days of spontaneous differentiation. However, the structure of the fitted tree suggested three main branches: days 0–2, 3–5, and 6–7. We labeled these branches as “pluripotent”, “intermediate”, and “differentiated”. With these three classes, the simplified decision tree yielded 87% validation accuracy in the prediction of the iPSC state from seven metabolic features. Predictor importance yielded 37% for *m/z* 885.6, 24% for *m/z* 687.5, 20% for *m/z* 940.6, and 19% for *m/z* 778.5.

### PLS Discriminant Analysis Reveals Spatial Correlation of Phospholipid Abundance and Pluripotency Markers

**Figure 3:**
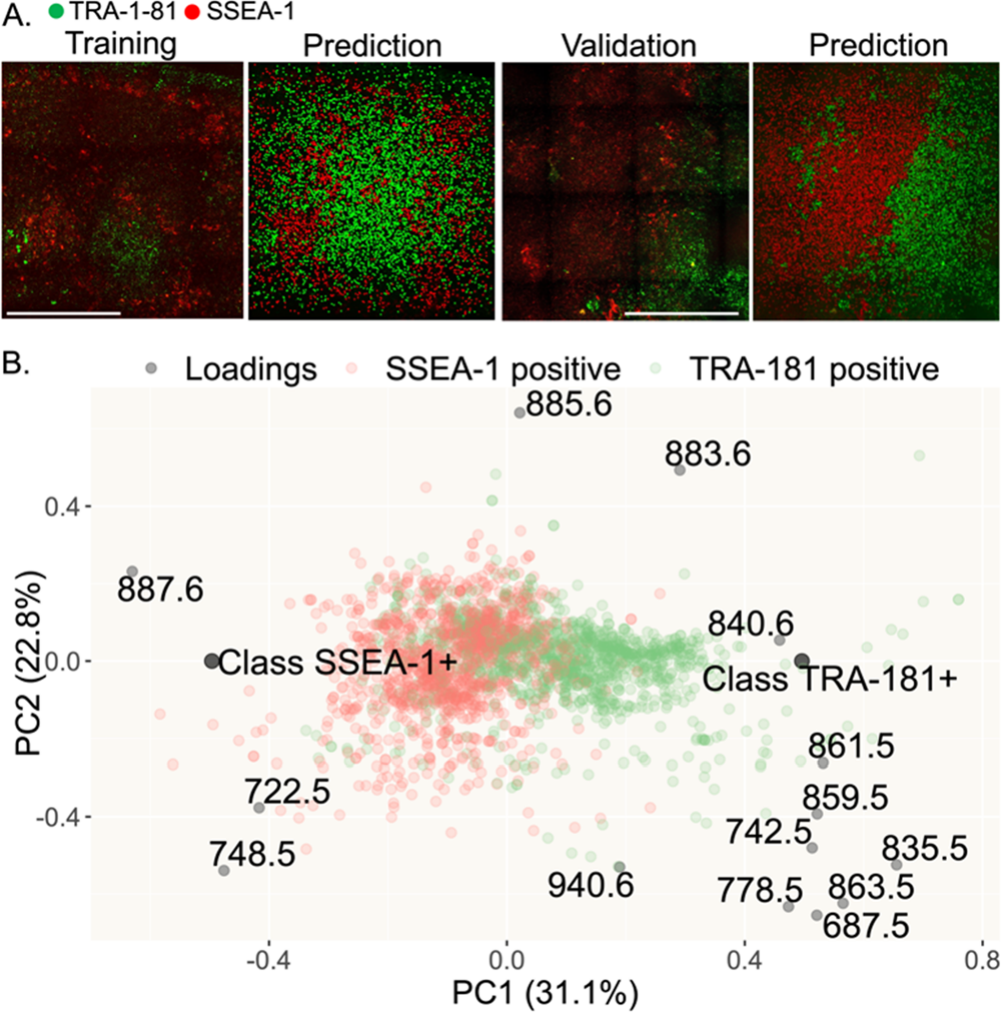
Phospholipid abundances and pluripotency markers are spatially correlated. (A) Training and validation confocal images of day 6 of spontaneous differentiation and their predicted pluripotency labels. Green color labels pluripotent cells, and red color labels differentiated cells. Scale bars: 1 mm. (B) Biplot of the PLS-DA model used to discriminate between SSEA-1+ and TRA-181+ populations based on the cells’ phospholipid abundance with 90% validation accuracy.

To associate the pluripotency status of iPSCs in a colony with their metabolic signatures, we analyzed the spatial correlation of *m/z* features with the fluorescent pluripotency labels in the imaged colonies. We selected day 6 of spontaneous differentiation for analysis because the cell colony was exhibiting significant expression of both TRA-181 and SSEA-1 pluripotency markers. None of the days showed the expression of NCAM-1. Cells in the training sample (Figure 3a, left side) were labeled as TRA-181-positive or SSEA-1-positive based on *k*-means clustering (*K* = 2) of the respective fluorescence intensities. We used an experimental replicate of day 6 as the validation dataset (Figure 3a, right side). We trained a partial least-squares discriminant analysis (PLS-DA) classifier (Figure 3b), and, after variable trimming, the validation accuracy was 90%. The predicted cell labels are plotted in Figure 3a alongside the original confocal images. A cluster of variables correlated with TRA-181-positive (pluripotent) cell population included *m/z* 742.5, 778.53, 861.5, 863.5, and 940.6, in agreement with the decline in their abundance with the differentiation time shown in Figure 2a. Similarly, *m/z* 722.5 and 748.5 were correlated with SSEA-1-positive (differentiated) cell population, in agreement with their increase with the differentiation time. It is worth noting that in our experiments we observed TRA-181 expression lagging behind Oct4 expression, showing a higher percentage of pluripotent cells during live imaging compared to the flow cytometry measurements of Oct4 expression. This highlights the shortcomings of the current live pluripotency markers such as TRA-181 and emphasizes the need for novel targets to be utilized for live pluripotency monitoring.

### Inhibition of Phosphatidylethanolamine *N*-Methyltransferase Prolongs Pluripotency during Spontaneous Differentiation

**Figure 4:**
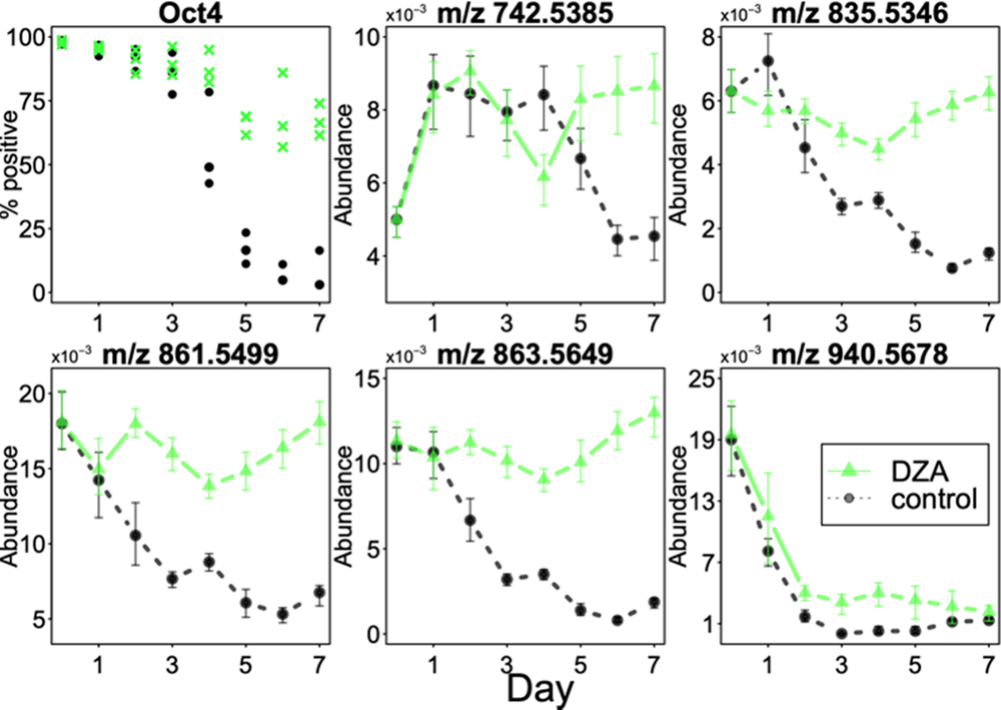
Continuous exposure to 3-deazaadenosine (DZA) promotes pluripotency maintenance following the perturbation of phospholipid abundances. More than 50% of population maintained Oct4 expression in the DZA-exposed sample in three independent experiments (top left). The MALDI FTICR MS analysis of control and DZA-exposed samples revealed that several phospholipids that decline with the differentiation time in the control experiment maintain their abundances in the DZA experiment, correlating with the Oct4 expression. Data points represent the average *m/z* abundances per image, and error bars show 25th and 75th percentiles.

We annotated as many detected lipids as possible through MS/MS experiments and accurate mass measurements (Table S2) to relate the metabolic features with biological functions. Several phospholipids with abundance changes associated with the differentiation process were annotated as phosphatidylethanolamines (PEs). With the previous studies suggesting that phosphatidylcholines (PCs) are involved in differentiation,^16^ we disrupted the PE-to-PC conversion pathway by inhibiting PEMT by the addition of 50 μM of 3-deazaadenosine (DZA) to the differentiation media throughout all 7 days of differentiation. We observed via flow cytometry (Figure 4, top left panel) that continuous DZA exposure prevents Oct4 expression loss with differentiation. To reveal the changes in phospholipid abundances following this perturbation, we evaluated additional eight iPSC colony samples, one for each day of spontaneous differentiation with a constant DZA exposure. As we did not observe any changes in the spatial organization of pluripotency marker expression, we conducted mass spectrometry analysis using MALDI FTICR imaging, with a pixel size of 25 μm and ultrahigh mass resolution, to better track individual lipid species. In these experiments, we did not detect changes in PC abundances. However, we observed an increase in *m/z* 742.5385 (PE 36:2) in days 5, 6, and 7, correlating with the changes in Oct4 expression in control versus the DZA-exposed sample. The most dramatic ion abundance increases compared to the control were for *m/z* 835.5346 (PI 34:1), 861.5499 (PI 36:2), and 863.5649 (PI 36:1), highlighting once again that changes in PI phospholipids precede changes in pluripotency transcription factors (Figure 4). MicroRNAs have been reported as master metabolic controllers of naïve to primed ESC state and reprogramming to iPSCs and potentially alter lipid-synthesizing and lipid-catalyzing enzyme expression levels in advance of differentiation in iPSCs.^18,19^ While the unknown species at *m/z* 940.5678 did not match the changes in Oct4 expression that occur with DZA inhibition, it could reflect the underlying spectrum of cell pluripotency status including epigenetic changes that precede the drop in Oct4 expression. This lipid species resisted all attempts of structural annotation due to its comparatively lower signal-to-noise ratio, even with some of the most modern MALDI imaging MS instrumentation available and extensive MS/MS analysis attempts.

### Inhibition of Phosphatidylinositol 3-Kinase Results in Increased NCAM-1 Expression and Changes in Colony Spatial Organization

**Figure 5:**
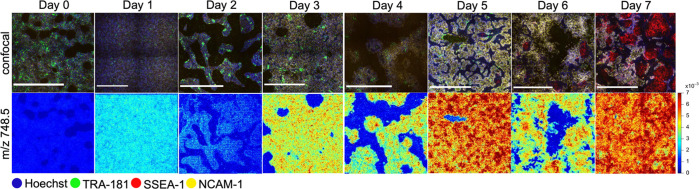
Phosphatidylinositol 3-kinase inhibition changes colony organization as observed by phospholipid abundances via MALDI imaging. Top row––confocal images of iPSC colonies undergoing differentiation for 7 days with the addition of 100 μM LY294002 on day 0; blue is Hoechst staining, green is TRA-181, red is SSEA-1, and yellow is NCAM-1. Bottom row shows the corresponding MALDI ion images for *m/z* 748.5, with blue color representing the low peak abundance and red representing high abundance.

The *m/z* 835.5346, 861.5499, and 863.5649 species detected by MALDI FTICR MS belong to the phosphatidylinositol (PI) family (Table 1). To further clarify the importance of PI cycling in the differentiation process, we conducted a series of experiments in which we initiated differentiation while inhibiting phosphatidylinositol 3-kinase with LY294002. We characterized eight iPSC colony samples, one for each day of spontaneous differentiation, with a low inhibitor concentration of 35 μM (Figure S4a) and another eight samples with a high inhibitor concentration of 100 μM (Figure 5). While performing confocal microscopy imaging on these samples, we observed a dose-dependent increase in NCAM-1 expression compared to controls (Figure S3c), as well as changes in the spatial organization of NCAM-1- and SSEA-1-positive cells (Figures 5 and S5). When comparing the phospholipid abundance trajectories between the three conditions (control, 35, and 100 μM inhibition, Figure S4b), we observed the absolute values of trajectories’ slopes increase in a dose-dependent manner for several PI family members (*m/z* 859.5, 863.5, 883.6, and 911.5). The representative *m/z* 748.5 ion showed consistent growth in all three conditions as well as a spatial correlation with SSEA-1 expression and a strong anticorrelation with NCAM-1 expression (Figures 5 and S4a). The distinctive trajectories of *m/z* 778.5 and 940.6 were conserved with PI 3-kinase inhibition (Figure S4b). We observed that cells remained more pluripotent on the edge of the colony over the course of differentiation from immunocytochemistry performed on iPSC colonies stained with Oct4 for pluripotency, Otx2 for ectoderm differentiation, and Pax6 for neural lineage (Figure S6). To compare the phospholipid abundances in the center and on the edge of the colony, we divided the cells into seven groups based on their location in the colony and calculated the average *m/z* ion abundances for seven different distances from the edge. Some phospholipids (e.g., *m/z* 722.5 and 748.5) gradually increased in abundance with the distance from the edge and some gradually decreased (e.g., *m/z* 778.5 and 940.6), mostly consistent with the previously shown pluripotency correlation.

**Table 1:**
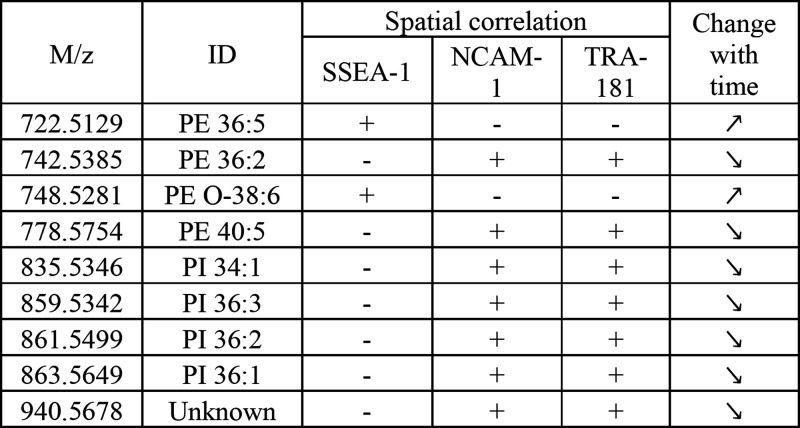
Summary of Lipid Ions Consistently Correlating with the Cell Fate^a^

a See Table S2 for expanded annotation.

**Figure 6:**
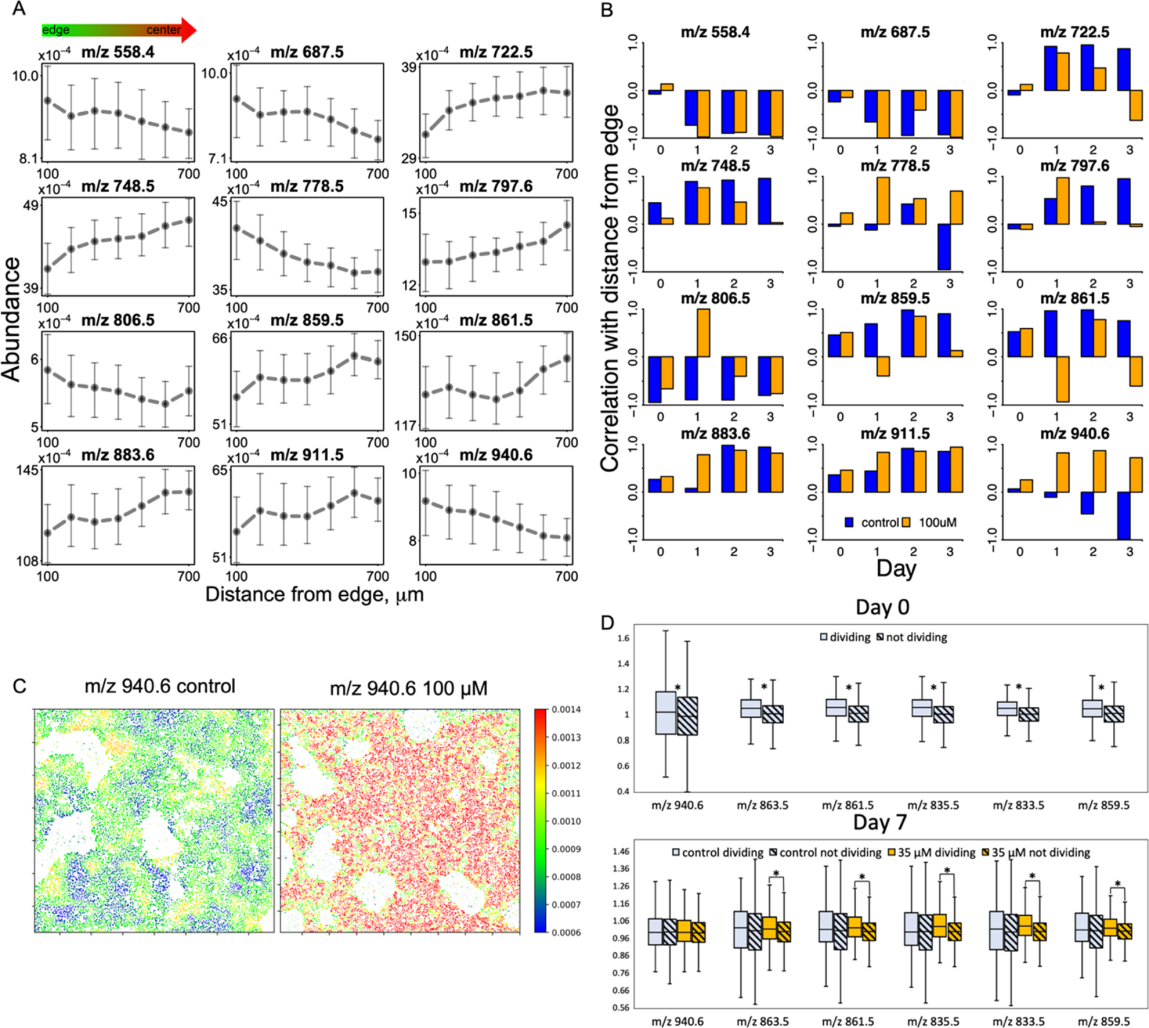
Phospholipid abundances change with colony and cell morphology. (A) Mean phospholipid abundances on day 3 of controlled spontaneous differentiation change with the distance from the edge of the colony. Points represent mean values within the 100 μm distance range, and error bars show 25th and 75th percentiles. (B) Correlation of phospholipid abundances with edge distance changes with days of differentiation and with LY294002 addition. (C) Spatial distribution of *m/z* 940.6 abundance in day 3 of the control experiment shows increased signal on the edge of the colony in contrast with high LY294002 dose experiment, which shows decreased signal on the edge of the colony. (D) Differences in neighbor-relative lipid abundances in dividing vs nondividing cells. Top: presented lipids are significantly more abundant in dividing cells on day 0. Bottom: by day 7, control samples stop exhibiting significant differences in lipid abundances, while differences in PI 3-kinase-inhibited samples are still significant. Shaded boxes represent nondividing cells. Box boundaries show 25th and 75th percentiles, middle line shows median, and whiskers show minimum and maximum values. Asterisks show statistical significance in median differences; *p* value < 0.05.

Examples of such trends for day 3 in the control experiment are shown in Figure 6a. Immunocytochemistry images (Figure S6a) suggested that the difference between the edge and the center of the colony became more prominent with the overall colony differentiation, consistent with some phospholipids showing a higher correlation with the edge distance in the later days of differentiation and little correlation on day 0 (Figure 6b). We also observed a correlation “flip” for some lipids (e.g., *m/z* 940.6) in the PI 3-kinase-inhibited experiment (Figure 6b). While this trend is not reflected in the immunocytochemistry images of days 0–3 of the PI 3-kinase-inhibited differentiation, day 4 starts to reveal a mixed Oct4/Otx2 pattern, with days 5 and 6 in the 100 μM LY294002 experiment showing a reversed spatial pattern of pluripotency, with increased Otx2 expression on the edge of the colony and Oct4 expression in the center (Figure S6b). PI 3-kinase activates Akt which is involved in cell migration and mTOR pathways, perhaps explaining the formation of spatial clusters of lineage markers in an edge-independent way in PI 3-kinase-inhibited colonies––possibly, the spontaneous centers of differentiation do not migrate out into the colony, creating a more localized progeny.

### Phospholipid Abundances Vary Based on the Proliferative Status of Cells

Because Akt signaling is strongly related to cell proliferation, we hypothesized that cells undergoing mitosis would reflect the differences in PI signatures. To find metabolic signatures corresponding to mitotic cells, we developed a *k*-means clustering algorithm to distinguish the cells undergoing mitosis by their nuclear morphology and the brightness of the Hoechst stain. To test the algorithm, we manually annotated dividing nuclei in a small ROI; the algorithm yielded 98.8% prediction accuracy. Next, using the overlaid and aligned MALDI MS images, we associated the cell’s proliferative status to its lipidomic signature. As this task required precise single-cell comparison, we used a neighbor-relative abundance metric to account for potential unevenness of the background. Finally, we compared the ion abundances between the dividing and nondividing cells on day 0 of differentiation (Figure 6d, top). We observed higher neighbor-relative abundances from *m/z* 835.5, 861.5, 863.5, and 940.6 in dividing cells, which is consistent with these ions’ previous correlations with pluripotency due to the faster cell cycle of pluripotent cells. By day 7 of the control differentiation, these differences disappear; however, they are maintained in PI 3-kinase-inhibited differentiation (Figure 6d, bottom). As the PI3K/Akt pathway is involved in iPSC proliferation and differentiation,^20^ possibly, cells that continue to divide despite PI 3-kinase inhibition have a more contrasting phenotype compared to the dividing cells in the control condition.

### Phospholipid Profiles Reveal a Bifurcation in Cell Lineage Specification upon PI 3-Kinase Inhibition

**Figure 7:**
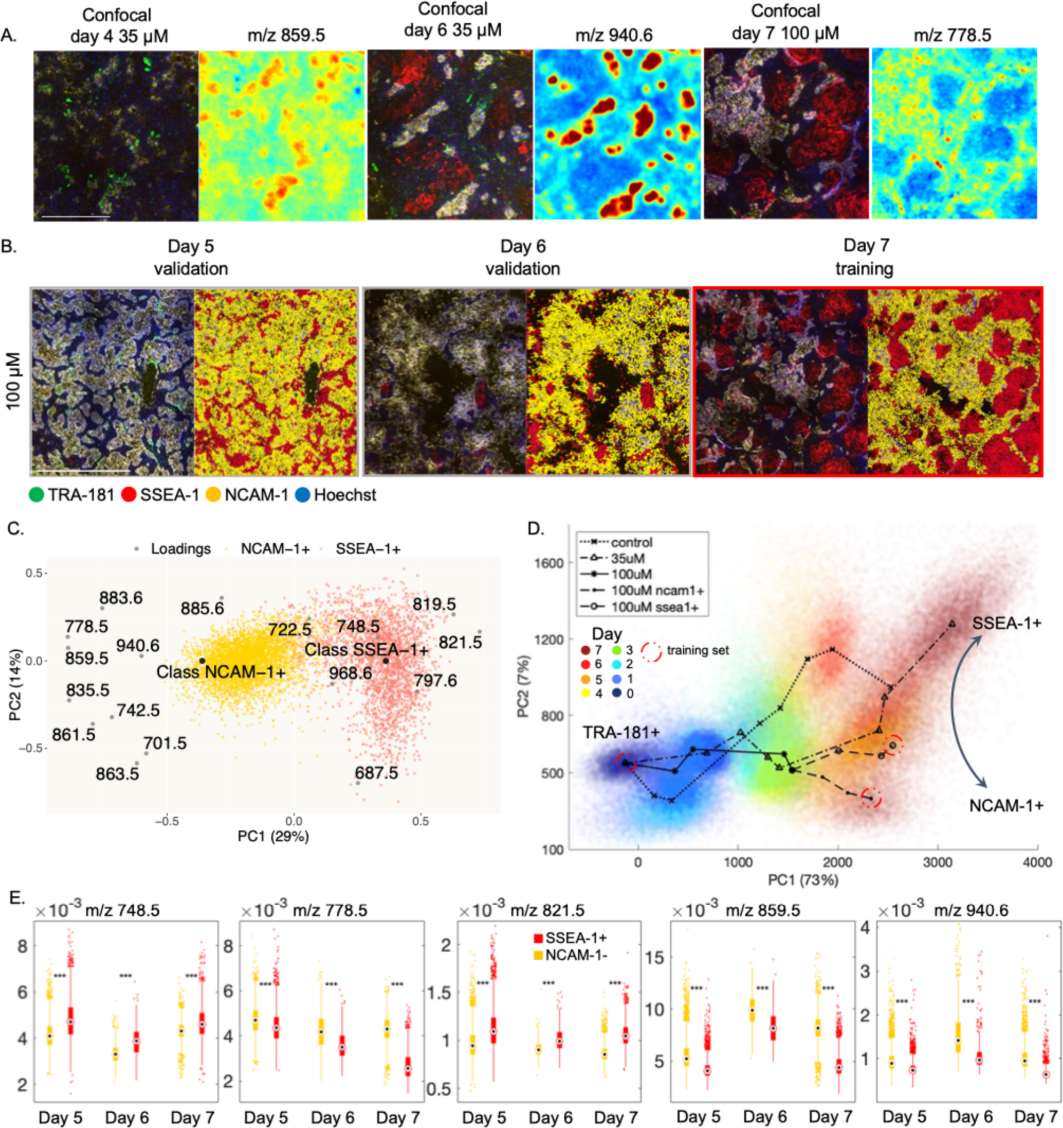
Changes in the spatial organization of pluripotency markers and phospholipids with PI 3-kinase inhibition. **(**A) Examples of ionic species correlating with cell lineage markers. Colors in confocal images are as follows: blue is Hoechst, green is TRA-181, red is SSEA-1, and yellow is NCAM-1. MALDI ion images are pseudo-colored, with blue showing low abundances and red showing high abundances. Scale bar: 0.5 mm. (B) Confocal images of days 5–7 of spontaneous differentiation with 100 μM of LY294002 and their predicted lineage labels. Red color labels: SSEA-1+ cells, and yellow color: NCAM-1+ cells. Day 7 was used as a training set and days 5 and 6 as validation sets (80 and 90% accuracies). Scale bar: 1 mm. (C) Biplot of the PLS-DA model used to discriminate between the cell populations in Figure 7b. (D) Principal component space created by training a PLS-DA model with three main populations: pluripotent cells (day 0) and NCAM-1+ and SSEA-1+ cells of day 7 (100 μM of LY294002). The rest of the data from all three experiments were projected into this principal component space. Red color indicates the later days of differentiation, and blue color indicates early days. (E) Boxplots comparing the abundances of the featured phospholipids between NCAM-1+ and SSEA-1+ populations. Triple asterisks show statistical significance, and two-tailed t test *p* value < 0.001.

Along with the increased neural lineage specification showed by NCAM-1 expression, PI-3 kinase inhibition resulted in distinct spatial clustering of cells with similar cell fate marker expression (Figure 7a). Such clustering further highlighted the spatial correlation of certain phospholipids and cell lineage markers. The ion at *m/z* 748.5 (PE O-38:6) was strongly anticorrelated with NCAM-1 expression and correlated with SSEA-1 expression, as well as consistently increasing with differentiation time in all three experiments; this shows that PE O-38:6 is consistently correlated with iPSC differentiation, both spatially and temporally. The unknown lipid species at *m/z* 940.6 strongly correlated with NCAM-1 expression, along with other metabolic markers that correlated with pluripotency in previous experiments. These findings suggest that the observed NCAM-1-positive cell population is metabolically closer to the pluripotent state than the rest of the colony, which we confirmed by the immunocytochemistry images showing the co-expression of NCAM-1 and Oct4 in PI 3-kinase-inhibited experiments (Figure S5). To quantify the described spatial correlations of lipid abundances and fluorescent labels and identify metabolic signatures corresponding to newly emerging cell populations, we trained a PLS-DA classifier using the last 3 days of differentiation to determine if the metabolic changes during PI3K inhibition bifurcate in a predictable manner. Inhibition with 100 μM of LY294002 resulted in two distinct cell populations: SSEA-1+ and NCAM-1+, and no TRA-181-positive cells (Figure 7b), suggesting that high doses of the inhibitor drive cells toward the neural lineage specification. As day 7 had equal representation of both populations, we used it as a training set and withheld day 5 and day 6 as validation sets. After variable trimming, the training set yielded 95% accuracy; day 5 and day 6 yielded 80 and 90% accuracy, respectively (Figure 7b). The PLS-DA biplot (Figure 7c) shows distinct SSEA-1+ and NCAM-1+ correlated clusters of both observations (scores) and variables (loadings). These clusters of variables represent distinct lipid signatures of the two populations: the SSEA-1+ population had increased abundances of *m/z* 722.5, 748.5, 819.5, and 821.5, while the NCAM-1+ population had increased abundances of PI lipids (*m/z* 859.5, 861.5, 863.5, 883.5, and 885.6), along with *m/z* 778.5 and 940.6. Quantitative differences in featured phospholipid abundances between the two cell lineages are shown in Figure 7e. Notably, a divergence between the populations is increasing with the differentiation time, as can be seen from the changes in abundance of *m/z* 778.5. Most of the lipids correlating with the NCAM-1+ population were marked as correlated with TRA-181 expression, which is consistent with the NCAM-1+ population correlating with Oct4 expression in Figure S5. NCAM-1+ and TRA-181+ populations, although similar, do not show the same expression pattern and thus do not possess the same phenotype. This may explain any inconsistencies between Table 1 and Figure 7c. To summarize the relationships between our experiments, we selected three main observed phenotypes as the training set for the PLS-DA model: the SSEA-1+ and NCAM-1+ populations from day 7 of the 100 μM condition and cells from day 0 as a pluripotent population.

Next, we projected all the data into this principal component space (Figure 7d). We observed a correlation of day of differentiation and PC1, indicating that PC1 represents the time in the principal component space. We also observed the divergence of NCAM-1+ and SSEA-1+, suggesting that PC2 is representative of the cell fate. A compilation of our findings is provided for the phospholipid species consistently connected to cell fate throughout our analysis (Table 1).

## Conclusions

Induced pluripotent stem cells are emerging as a powerful regenerative medicine tool for the creation of patient-specific tissues for autologous transplantation.^21^ Investigating the mechanisms underlying the initial loss of pluripotency in iPSCs is desirable for revealing early quality control targets, preventing the wasting of time and resources on a batch bound to fail.^22,23^ Our multimodal imaging co-registration pipeline produced robust datasets that tied together cells’ location, morphology, cell fate surface markers, and metabolic profile. Multivariate analysis performed on these datasets consistently illustrated the predictive power of metabolic data, allowing for the accurate prediction of priming for differentiation or a cell’s surface marker expression as well as being informative about the cell’s proliferative status and location within the colony. This approach allowed us to establish robust and predictable early metabolic markers of pluripotency loss during spontaneous differentiation; because these changes occur earlier than the decline in Oct4 expression, these phospholipids hold potential as novel quality control targets in a cell manufacturing setting. Our analysis also informed multivariate trajectories revealing divergent metabolic cell fate, which could be useful in regenerative medicine applications by identifying key windows of differentiation in which lineage specification can be manipulated and/or corrected. Future work includes further investigation of the role of phosphatidylinositols in the self-organization of 3D iPSC organoids and under directed differentiation protocols. Because many of the phospholipids identified in our analysis are involved in lipid bilayer structure and function,^24^ elucidation of additional label-free morphological features associated with lipid properties that reflect the dynamic metabolic signatures discovered here is a potential avenue for nondestructive monitoring in the cell manufacturing of iPSC-derived tissues.

## Supporting Information Available

List of the used materials and reagents; iPSC culture maintenance protocol; flow cytometry protocol; immunocytochemistry protocol; computational methods for nuclei segmentation and cell metrics calculations; details on machine learning analyses used; temporal and spatial changes induced by PI3K inhibition; immunocytochemistry images of control and PI3K-inhibited iPSC colonies undergoing differentiation; and dividing cells’ manual annotation and validation results (PDF)

## Notes

The co-registration and segmentation codes with data samples can be found at our lab’s GitHub (https://github.com/kemplab/coregistration_gui).
