# Supplementary material for: Mass Spectrometry Imaging Reveals Early Metabolic Priming of Cell Lineage in Differentiating Human-Induced Pluripotent Stem Cells
Source: Anal Chem. 2023 Mar 10;95(11):4880–8. doi: 10.1021/acs.analchem.2c04416 (PMC10034746; doi:10.1021/acs.analchem.2c04416)
Supplement: Supplementary file 1 — ac2c04416_si_001.pdf [file ac2c04416_si_001.pdf]

Supporting information

Mass spectrometry imaging reveals early metabolic priming of cell lineage in differentiating human induced pluripotent stem cells

Arina A. Nikitina[1], Alexandria Van Grouw[2], Tanya Roysam[3], Danning Huang[2], Facundo M. Fernández[2,4], Melissa L. Kemp[3,4*]

[1]School of Biological Sciences, Georgia Institute of Technology, Atlanta, Georgia, 30332, United States
[2]School of Chemistry and Biochemistry, Georgia Institute of Technology, Atlanta, Georgia, 30332, United States
[3]The Wallace H. Coulter Department of Biomedical Engineering, Georgia Institute of Technology and Emory University, Atlanta, Georgia, 30332, United States
[4]Petit Institute of Bioengineering and Biosciences, Georgia Institute of Technology, Atlanta, Georgia, 30332, United States
* Corresponding author, melissa.kemp@bme.gatech.edu

Table of Contents:

Supplemental Methods
- S1. Materials
- S2. Cell Culture
- S3. Flow Cytometry
- S4. Immunohistochemistry
- S5. Nuclei Segmentation
- S6. Metrics
- S7. Machine Learning

Figure S1. Custom-made silicone 8-well wall adhered to an ITO slide used for cell culture
Figure S2. Partial least-squares regression (PLSR) of the day of differentiation against lipid abundances
Figure S3. Surface markers fluorescence intensity on a cell-by-cell basis
Figure S4. Temporal and spatial changes induced by phosphatidylinositol 3-kinase inhibition
Figure S5. Ncam1 and Oct4 spatial expression in iPSC colonies are altered with LY294002
Figure S6. Immunocytochemistry images of the 8-day spontaneous differentiation for control and PI 3-kinase inhibited conditions
Figure S7. Dividing cells manual annotation and validation results
Figure S8. Averaged mass spectra examples from images collected using MALDI-TOF of iPSC colonies
Figure S9. Representative blank spectra collected on Bruker Rapiflex MALDI TOF and Bruker SolariX MALDI FTICR
Table S1. Summary of all experimental conditions tested
Table S2. Lipid annotations for 16 selected features found in all 3 spontaneous differentiation experiments

S1. Materials.

Acetonitrile (LC-MS grade), ammonium formate, and norharmane (98%) were purchased from Fisher Chemical (Pittsburgh, PA, USA). Laboratory grade Triton™ X-100, 3-Deazaadenosine, acetone and red phosphorus (≥99.99% purity) were purchased from Sigma Aldrich (Sigma-Aldrich Corporation, St. Louis, MO, USA). Ultrapure water with 18.2 MΩ·cm resistivity (Barnstead Nanopure UV ultrapure water system, USA) was used to prepare the ammonium formate buffer wash solution. Conductive ITO glass slides were purchased from Bruker Daltonics (Billerica, MA, USA). Dow SYLGARD™ 184 Silicone Encapsulant Clear Kit was purchased from Ellsworth Adhesives (Loganville, GA, USA). Anhydrous DMSO, Corning® Matrigel® Growth Factor Reduced (GFR) Basement Membrane Matrix (Phenol Red-Free, *LDEV-Free), KnockOut™ DMEM, Dulbecco's Phosphate-Buffered Saline, 10X with calcium and magnesium (DPBS), B-27™ Supplement (50X), Molecular Probes Hoechst 33342, and Donkey anti-Mouse IgG (H+L) Highly Cross-Adsorbed Alexa Fluor Plus 488 Secondary Antibody were purchased from Thermo Fisher Scientific (Waltham, MA, USA). MTeSR™ Plus (Basal Medium and 5X Supplement), Y-27632 RHO/ROCK pathway inhibitor, and accutase were purchased from Stemcell Technologies (Cambridge, MA, USA). RPMI 1640 was purchased from Caisson Labs (Smithfield, UT, USA). GloLIVE Human Pluripotent Stem Cell Live Cell Imaging Kit (catalog #NLLC2155R) including positive marker TRA-1-81 and negative marker SSEA-1 (301021), and Alexa Fluor 647-conjugated Human NCAM-1/CD56 (MC-480) live stain (catalog #FAB24081R-100UG) were purchased from R&D Systems (Minneapolis, MN, USA) as well as Human Three Germ Layer 3-Color Immunocytochemistry Kit (catalog #SC022) including Anti-Human Otx2 NL557-Conjugated Goat IgG. 6-Well Tissue Culture Plates were purchased from Celltreat (Pepperell, MA, USA). Paraformaldehyde Aqueous Solution was purchased from Electron Microscopy Sciences (Hatfield, PA, USA). Odyssey® PBS Blocking Buffer was purchased from LI-COR Biosciences (Linkoln, NE, USA). Mouse Anti-human Oct-3/4 (C-10) primary antibody (catalog #sc-5279) was purchased from Santa Cruz Biotechnology (Dallas, TX, USA). LY294002 was purchased from Cell Signaling Technology (Danvers, MA, USA). Mouse anti-Human Alexa Fluor 647-conjugated Pax6 antibody (O18 1330) was purchased from BD Biosciences (San Jose, CA, USA, catalog #562249).

S2. Cell culture

HiPSC WTC11 cells (Coriell Institute, catalog ID GM25256, sex: male) were grown in 6-well plates. Wells were coated with 1mL Matrigel (GFR in Knockout D-MEM, 1:100) per well and incubated overnight. Cells were fed 2 mL media per well daily (basal MTeSR Plus media + supplement, 4:1). During passage, 0.5 mL accutase was added to each well and incubated for 3 min. Cells were lifted and collected into a 15 mL tube with excess DPBS (~3x times accutase used). Cells were centrifuged at 1000 rpm for 5 minutes, supernatant was removed, and the pellet was lifted in 2 mL media with 2 μL Rock inhibitor. Cells were seeded at 100-200 K density in 2 mL media and 2 μL Rock inhibitor was added for the first day after passage, then cells were fed as usual.

S3. Flow cytometry

Eight control wells and 8 DZA-exposed wells underwent 0 to 7 days of spontaneous differentiation in 6-well plates, each well was reproduced 3 times. Cells were lifted with 0.5 mL accutase per a well of a 6-well plate, centrifuged at 1000 rpm for 5 minutes, supernatant was removed, and the pellet was lifted in 1 mL of 4% paraformaldehyde solution in PBS for cell fixation. After 10 min at room temperature cells were centrifuged again and resuspended in 1 mL of 0.3% Triton™ X-100 solution in PBS for permeabilization. After 15 min at room temperature cells were centrifuged and resuspended in 1 mL Odyssey Blocking Buffer for 1 hour at room temperature. Next, cells were centrifuged and resuspended in 1 mL Odyssey Blocking Buffer with 5 μL mouse

anti-human Oct4 primary antibody and were left at 4C overnight. After that, cells were centrifuged and resuspended in 1 mL PBS as a wash step. At this point, 0.5 μL of cells from Day 0 sample were set aside as a negative control. Next, cells were centrifuged and resuspended in 1 mL Odyssey Blocking Buffer with 1 μL anti-mouse Alexa Fluor Plus 488 secondary antibody for 30 minutes in the dark. Finally, after another wash step, cells were centrifuged and resuspended in 1 mL PBS and transferred to a FACS tube through the strainer cap. Samples were analyzed on BD FACSMelody™ Cell Sorter (BD Biosciences, San Jose, CA, USA) with excitation wavelength of 488 nm, detection in 515 nm-545 nm range. Data acquisition was performed with BD FACSChorus™ Software (BD Biosciences), data processing was performed with FlowJo™ (BD Biosciences). Oct4 gate was created so 99.9% of negative control fall into Oct4-negative category. Percentage of cells registered as Oct4-positive according to the gating was recorded for each sample.

S4. Immunocytochemistry

Eight control wells and 8 wells exposed to 100 μM LY294002 underwent 0 to 7 days of spontaneous differentiation on ITO-covered glass slides with a glued PDMS 8-well wall. Wells were washed with 100 μL of PBS each (wash step) and then fixed with 100 μL of 4% paraformaldehyde solution in PBS for 10 minutes. After 3 wash steps cells were permeabilized for 15 minutes with 100 μL of 0.3% Triton™ X-100 solution in PBS. After a wash step, cells were blocked with 100 μL of Odyssey Blocking Buffer for 1 hour at room temperature. Next, we diluted mouse anti-human Oct4 primary antibody at 1:200 ratio in Odyssey Blocking Buffer, added anti-human NL557-Conjugated Otx2 antibody at 1:100 ratio as well as anti-human Alexa Fluor 647-conjugated Pax6 antibody at 1:50 ratio. Cells were treated with 100 μL of antibody mixture for 1 hour in the dark. Next, after 3 wash steps cells were treated with 100 μL of Odyssey Blocking Buffer with Hoechst (1:1000) and anti-mouse Alexa Fluor Plus 488 secondary antibody (1:1000) for 30 minutes in the dark. Finally, after 3 wash steps 100 μL of PBS was added to each well and cells were imaged with Nikon UltraVIEW VoX W1 Spinning Disk Confocal with sCMOS camera at 10x magnification (0.65 mm/px), 100 ms exposure and 100% laser power for all wavelengths.

S5. Nuclei Segmentation

The confocal image stained with Hoechst was also used to segment nuclei to extract the abundance data at each *m/z* value of interest on a cell-by-cell basis along with the corresponding fluorescence intensities from the confocal image. First, a local threshold was applied on the image at window sizes ranging from a fourth of the image to twice the size of the largest cell (parameter provided by user); this was done to obtain the most comprehensive binary mask of the nuclei. Next, the segmentation algorithm utilizes a multiscale Laplacian of Gaussian (LoG) blob detection algorithm [1] implemented using OpenCV [2] for Python to find nuclei seeding points. The LoG of the image is computed for each radius in a user-provided range of nuclear radii. The LoG is found by applying a Gaussian filter with a standard deviation of $\sigma = {}^{r}\!/_{\sqrt{2}}$, where r is the radius in pixels, and subsequently finding the second spatial derivative of the image. This results in a range of images containing several local minima at the center of each blob; the intensity of the minimum at each blob corresponds with how closely the actual radius of the blob matches the σ parameter of the Gaussian filter. Following normalization of each image by multiplying it by σ^2, the minimum at each pixel across the stack of all calculated LoGs is taken. The center of each nucleus, or seed, can be found at the local minima of this resultant image, and applying the watershed transformation at these points on the binary mask yields the nuclear labels.

S6. Metrics.

Cell-to-cell connection distance is calculated by first finding a closest neighbor distance for each cell. Next, we clean these distances from outliers – remove all values that are higher than mean plus three standard deviations. Finally, the cell-to-cell connection distance value is assigned the maximum of the cleaned distances array. This approach guarantees that every non-outlier cell will have at least one cell within the cell-to-cell connection radius. The cells within that radius are called neighbors.

Neighbor-relative abundance was measured by first finding all neighbors for a cell of interest. Next, average abundance is calculated among the neighboring cells, and the self-value is divided by the average neighbor value.

To identify *dividing cells*, we segmented the nuclei images and calculated the following metrics for each cell: neighbor-relative Hoechst intensity, the area of the nucleus, and the distance to the nearest neighbor. A K-Means (K = 2) clustering algorithm from the scikit-learn library was then trained to classify the cells as either 'dividing' or 'not dividing' using those metrics. Out of the two resulting class centers, we designated a center with higher neighbor-relative Hoechst intensity, smaller area, and larger nearest neighbor distance to represent the dividing cells class. To estimate the classification accuracy, we manually annotated 3 1000×1000-pixel patches of Hoechst-stained colony image (Figure S7) and used the algorithm to predict cell labels, yielding an accuracy of 98.8%. Sensitivity of 72.5% and a positive predictive value of 96.3% showed that this method is much more prone to false negatives than false positives, which is preferrable when data has a disproportionately high number of negative datapoints. Statistical significance was determined by the two-tailed Mann-Whitney U test with significance threshold of $p\text{-value} < 0.05$.

To detect the edge of the colony we multiplied cell-to-cell connection distance by the user-provided value (default is 3) to expand the cell's neighborhood. Cells that are on the edge of a colony can be distinguished by having at least one side with no neighbors in its network. To determine if the cell is on the edge, a cell's personal neighborhood is represented as a series of vectors connecting the center node cell and each of its neighbors. Next, we sort these vectors by their angles, and if any difference between two consecutive angles is greater than $\pi/2$ radians, the cell is labelled as an "edge" cell. Next, the *edge distance* metric can be derived by finding the distance between a given cell and the edge cells and taking the minimum value.

S7. Machine learning.

A Shapiro-Wilk was performed to test that phospholipid abundance data is not distributed normally within one field of view. To train a classification tree, out of each day of differentiation we randomly selected 8000 cells and merged it into a training dataset of 64000 data points, with differentiation day number as a label and *m/z* values as features. We repeated this with a replica experiment obtaining a validation dataset of 64000 data points. Classification tree for the day of differentiation prediction was built using MATLAB built-in function *fitctree* with the number of tree splits limited by *MaxNumSplits* parameter set to 20. We used variable appending method for variable selection, iteratively adding those variables that increased the prediction accuracy on a validation dataset the most after being included into the analysis. After introducing first 5 variables this way the accuracy has stopped increasing, this indicated that these variables are the most predictive of the differentiation time point.

Prior to partial least squares discriminant analysis (PLS-DA), to determine which cells are TRA-1-81, SSEA-1 or NCAM-1 positive we first applied k-means clustering analysis to the corresponding extracted fluorescence intensities using MATLAB built-in function *kmeans* with number of clusters equal 2 for each live stain individually. For PLS-DA in control experiments we did not observe a significant number of NCAM-1 positive cells and thus focused on TRA-1-81 and SSEA-1 stains. Each cell was assigned a positive or a negative label for both stains (TRA-1-81+ or − , SSEA-1+ or −), as illustrated in Figure S3a. Next, we excluded double negative and double positive cells from the analysis as potential artifacts of staining and assigned all the remaining cells a pluripotent versus differentiated label, where pluripotent cells are positive in TRA-1-81 and negative in SSEA-1

and vice versa for the differentiated cells. Similarly, for the PI 3-kinase inhibited experiment (100 μM condition) we did not observe a significant number of TRA-1-81 positive cells and thus focused on NCAM-1 and SSEA-1 stains. Each cell was assigned a positive or a negative label for both stains (NCAM-1+ or − , SSEA-1+ or −), as illustrated in Figure S3b; double positives and double negatives were excluded from the subsequent analysis. After all cells received their lineage label, we used SIMCA® software (Sartorius AG, Göttingen, Germany) for PLS-DA to predict the labels using *m/z* values as features. To select the best predictors, we used variable trimming: iteratively removing every variable that reduced prediction accuracy on a validation dataset.

References

[1] Al-Kofahi, Y., Lassoued, W., Lee, W., Roysam, B. (2009). Improved automatic detection and segmentation of cell nuclei in histopathology images. IEEE Transactions on Biomedical Engineering, 57(4), 841-852.
[2] Bradski, G. (2000). The OpenCV Library. Dr. Dobb's Journal of Software Tools.

Figure S1. Custom-made silicone 8-well wall adhered to an ITO slide used for cell culture compatible with MALDI MSI. Location of each day of spontaneous differentiation is marked near the corresponding wells.

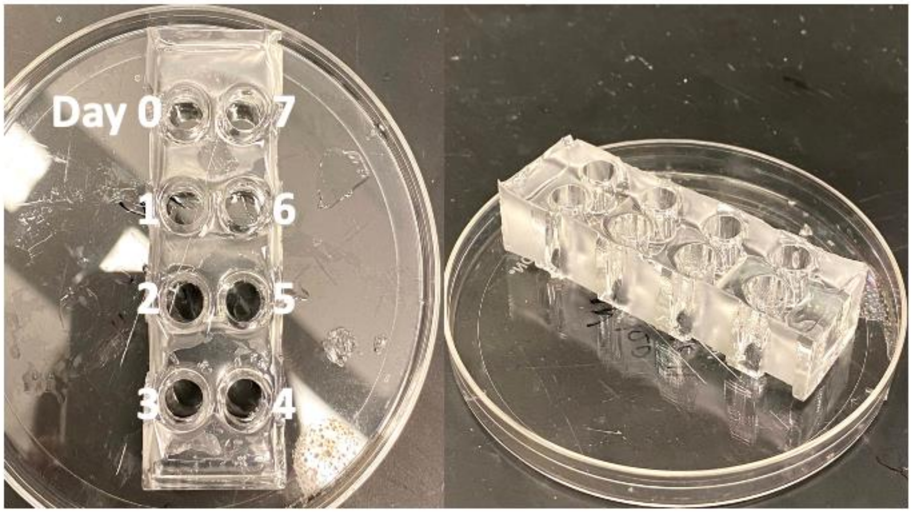

Figure S2. Partial least-squares regression (PLSR) of the day of differentiation against lipid abundances. Biplot on the left shows individual cells colored by the day of differentiation as scores and m/z values used in the PLSR as loadings. Only variables with a VIP score higher than 1 are shown on the biplot.

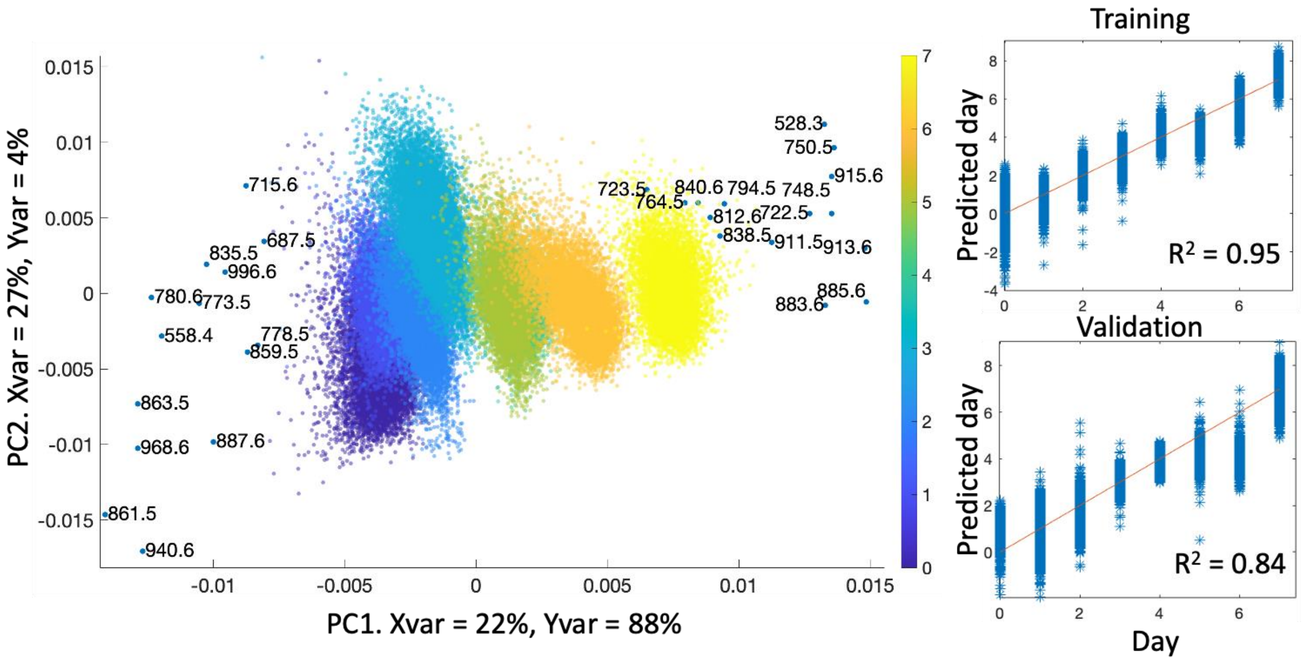

Figure S3. Surface markers fluorescence intensity on a cell-by-cell basis. A. Intensities of TRA-181 and SSEA-1 markers on day 6 of control experiment. Class boundaries determined through k-means clustering are 15.84 for SSEA-1 and 13.75 for TRA-181. B. Intensities of NCAM-1 and SSEA-1 markers on day 7 of PI 3-kinase inhibited experiment (100 μM dose). Class boundaries determined through k-means clustering are 15.51 for SSEA-1 and 14.94 for NCAM-1. Same class boundaries were applied to all days of the 100 μM condition. C. Histogram of NCAM-1 fluorescence intensity for days 5, 6, and 7 combined. Arrow shows the class boundary determined by k-means from day 7 of the 100 μM condition.

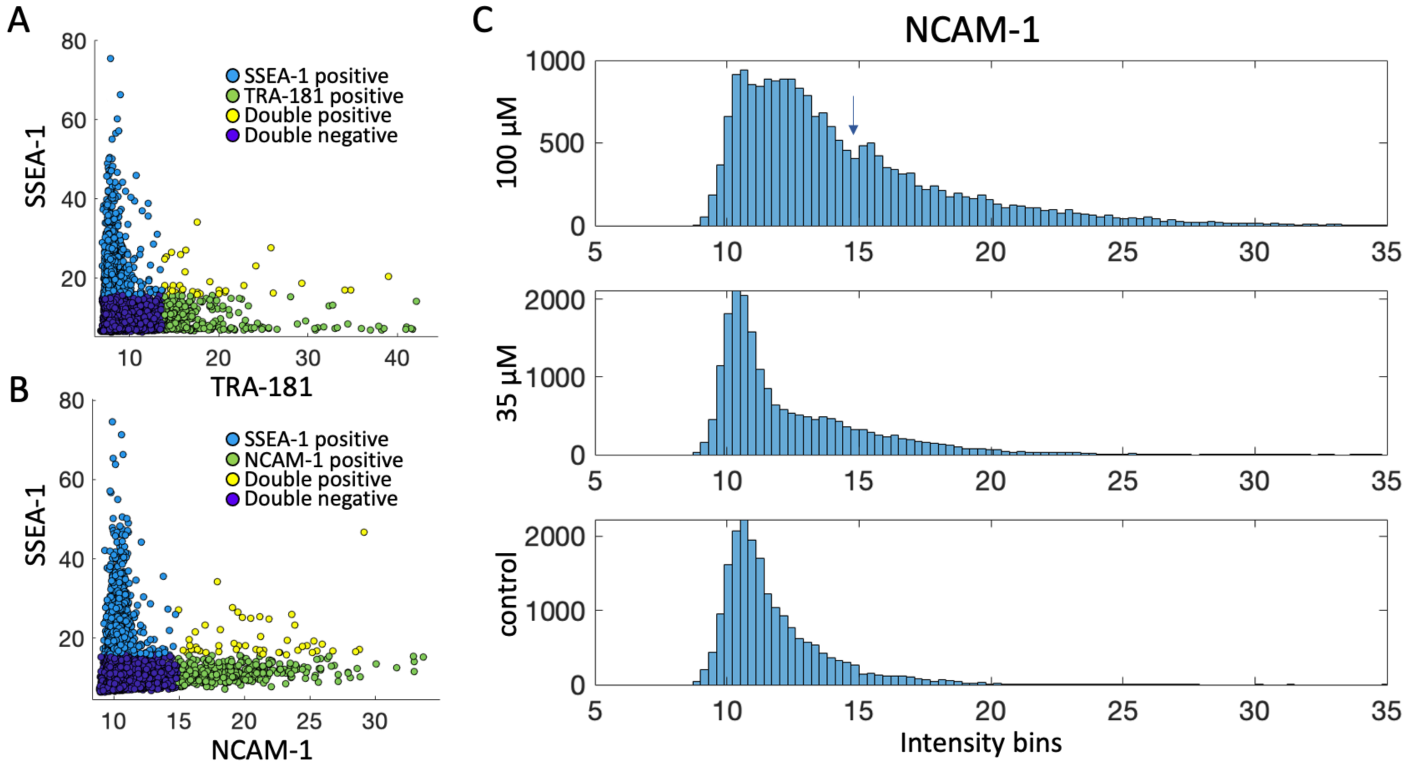

Figure S4. Temporal and spatial changes induced by phosphatidylinositol 3-kinase inhibition. A. Top row – confocal images of iPSC colonies undergoing differentiation for 7 days with addition of 35 μM LY294002 on day 0, blue is Hoechst staining, green is TRA-181, red is SSEA-1, yellow is NCAM-1. Bottom row shows corresponding MALDI ion images for m/z 748.5 with blue colors representing low peak abundance and red representing high abundance. B. Temporal changes in mean phospholipid abundance during spontaneous differentiation based on the LY294002 dose. Error bars show 25th and 75th percentiles.

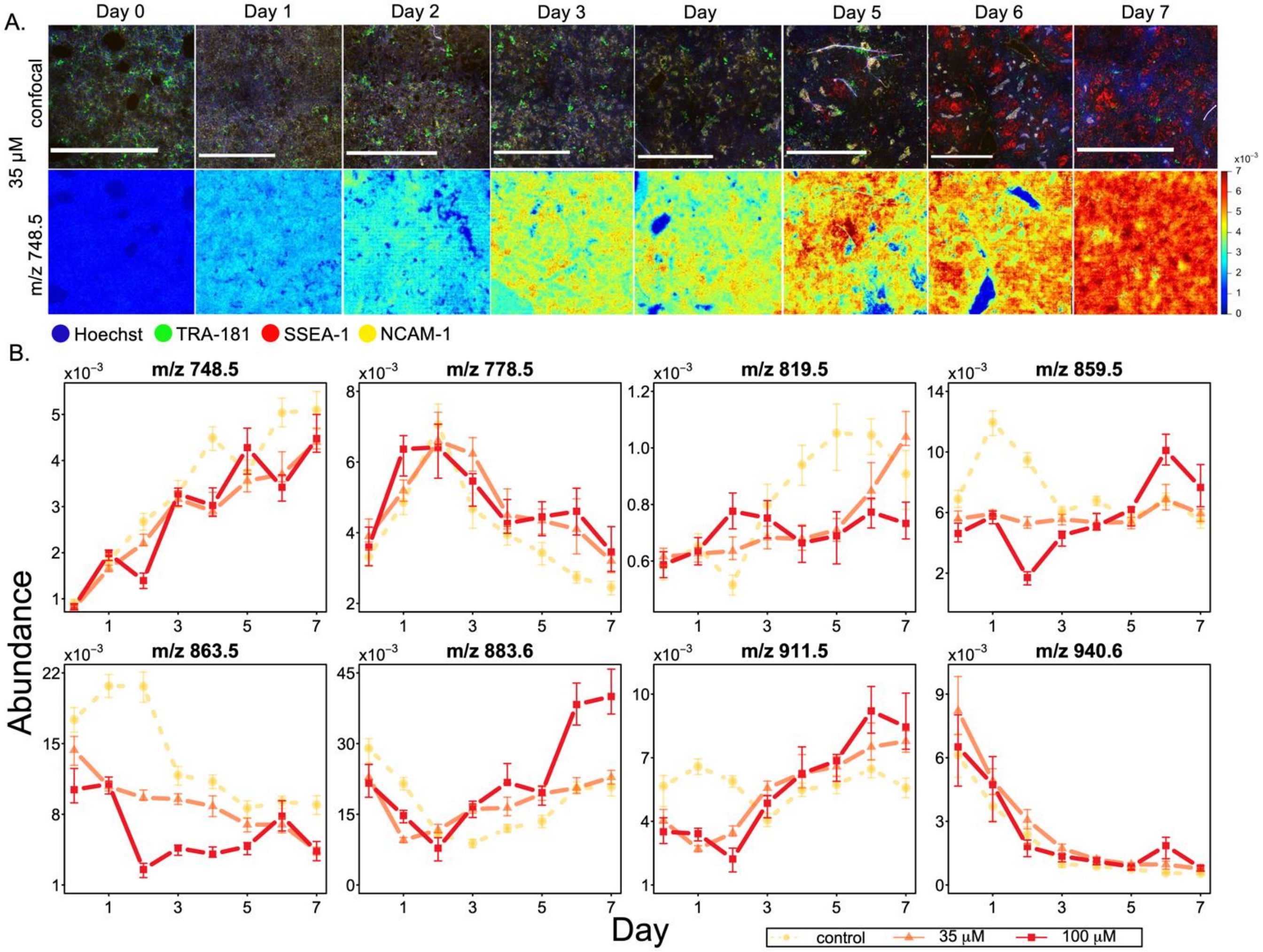

Figure S5. Ncam1 and Oct4 spatial expression in iPSC colonies are altered with LY294002. Edge-independent patterns of Ncam1/Oct4 expression from immunofluorescence imaging are observed as early as day 2 and 3 of PI 3-kinase inhibited differentiation with 35 μM or 100 μM LY294002, in contrast to the control differentiation with vehicle. Scalebars are 1 mm.

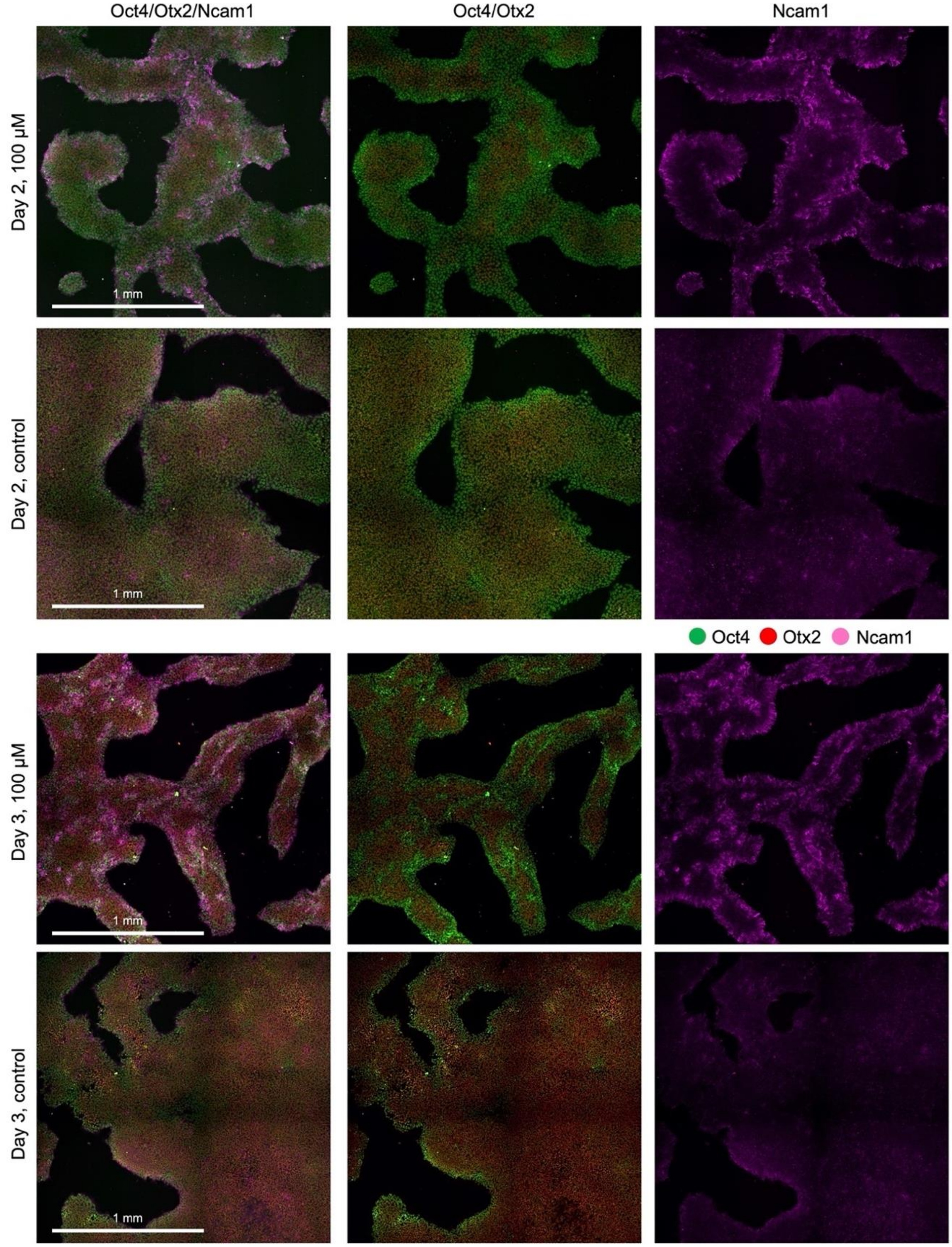

Figure S6. Immunocytochemistry images of the 8-day spontaneous differentiation for control and PI 3-kinase inhibited conditions. Cells were fixed and stained for Oct4 (green), Otx2 (red), and Pax6 (pink) in an independent set of experiments. A. Temporal changes of pluripotency markers expression in control versus PI 3-kinase inhibited samples. B. Difference in spatial patterns of pluripotency markers expression in control versus PI 3-kinase inhibited samples.

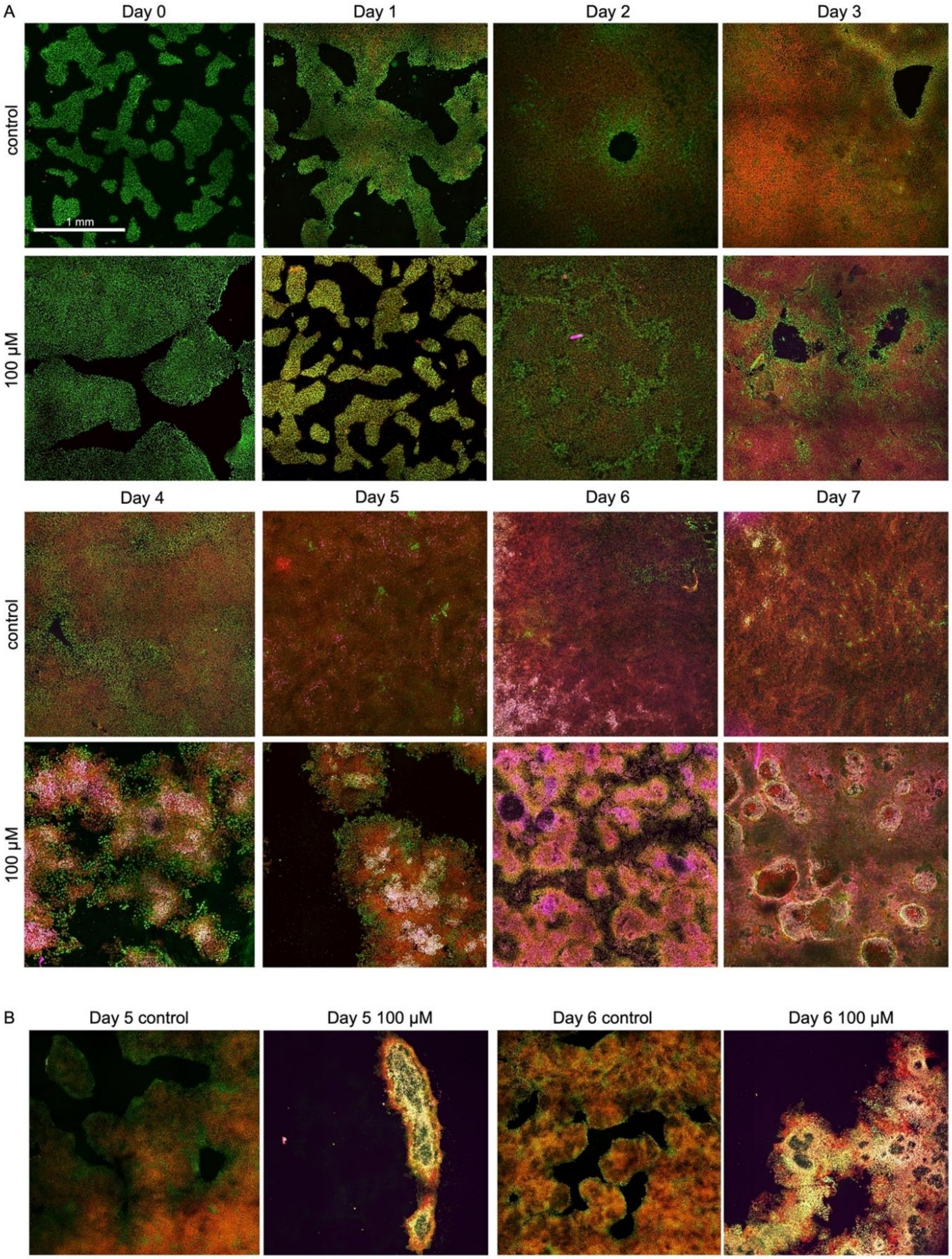

Figure S7. Dividing cells manual annotation and validation results. Circled nuclei were manually annotated as dividing (109 cells total). Segmentation algorithm detected a total of 2846 cells and neighbor-relative Hoechst intensity, area, and nearest neighbor distance were calculated for each cell. Using those metrics, K-means clustering predicted the dividing cells with accuracy of 98.8%, sensitivity of 72.5% and a positive predictive value of 96.3%.

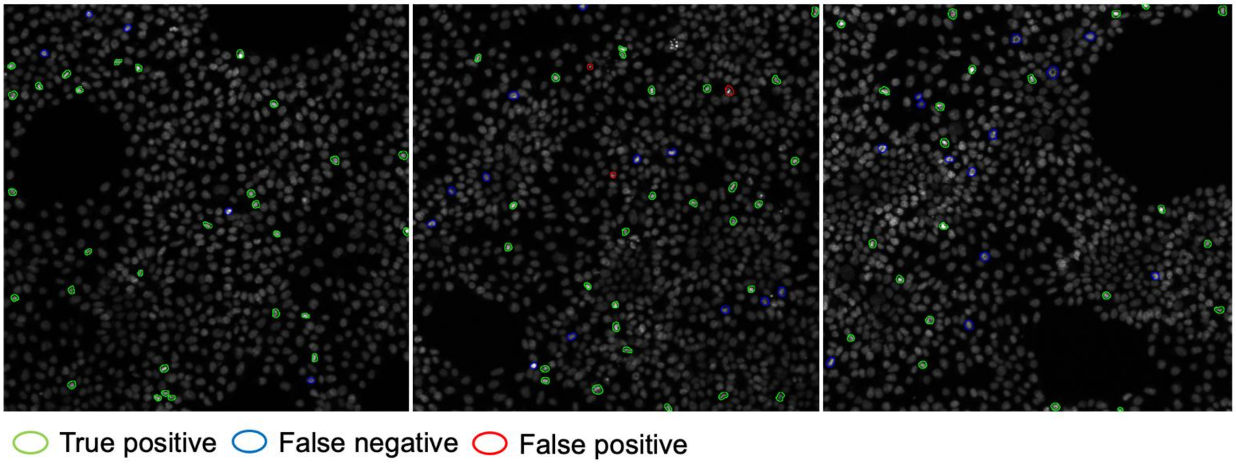

Figure S8. Averaged mass spectra examples from images collected using MALDI-TOF of iPSC colonies A) Control Day 0, B) Control Day 7, C) DZA treated Day 0, D) DZA treated Day 7, E) LY294002 35µM treated Day 0, F) LY294002 35µM treated Day 7, G) LY294002 100µM treated Day 0, H) LY294002 100µM treated Day 7.

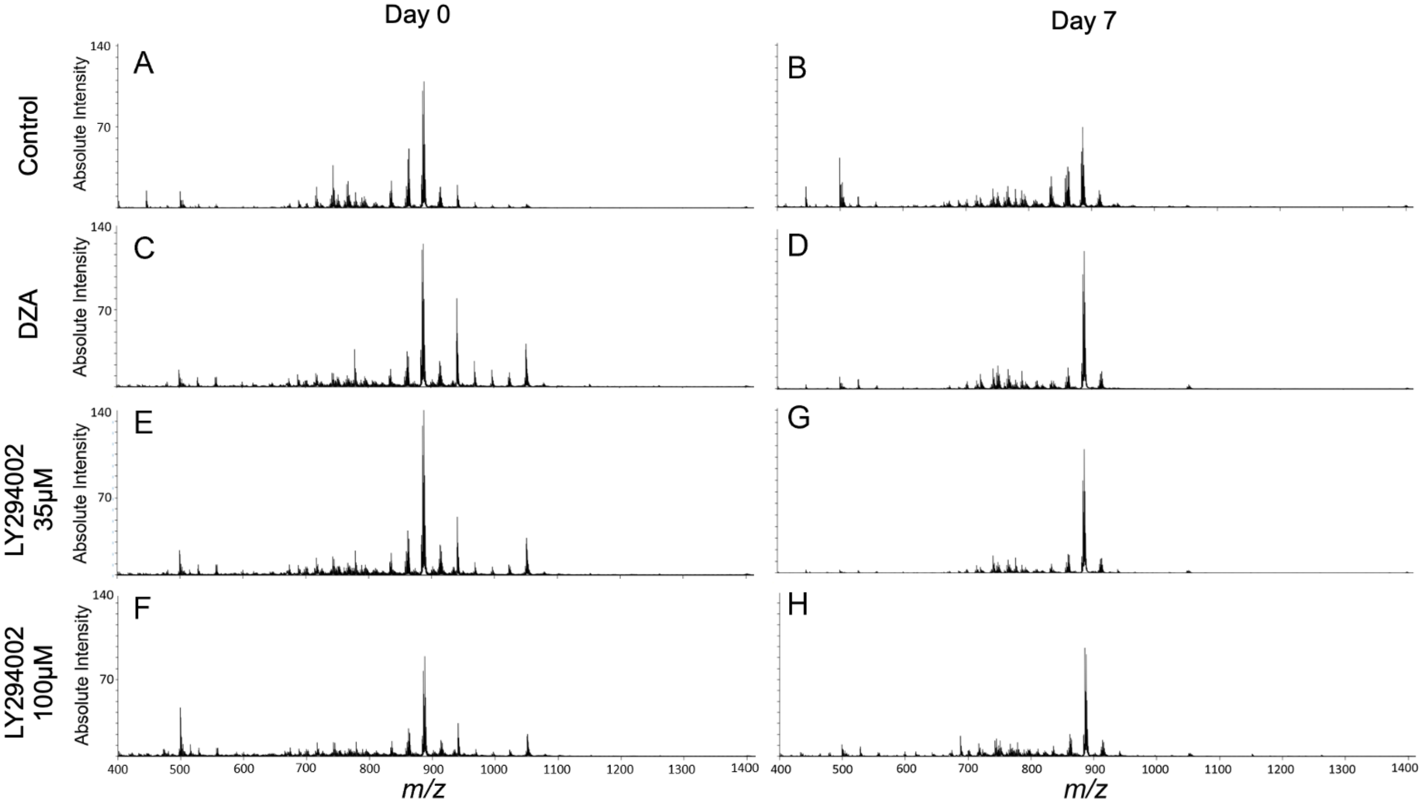

Figure S9. Representative blank spectra collected on A) Bruker Rapiflex MALDI TOF and B) Bruker SolariX MALDI FTICR. Blank spectra collected by sampling a small area on the experimental slide that does not contain cells but does contain norharmane matrix.

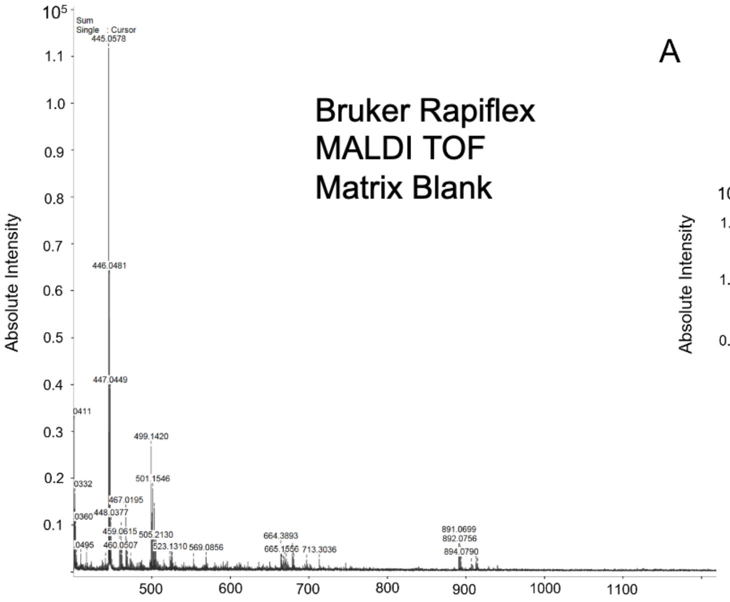

Table S1. Summary of all experimental conditions tested. Each slide had 8 wells, one well per day of spontaneous differentiation (days 0-7). Approximately 10-20×103 cells were co-registered for every well. If not indicated, matrix used is norharmane, deposition method is sublimation.

Experiment	Treatment	Method	Figure	Number of slides (replicas)
Spontaneous differentiation - control	None	MALDI TOF	1, 2, 3	2
		MALDI TOF, matrix: DAN, deposition: spray	Not shown	1
		MALDI TOF, matrix: DAN	Not shown	2
Spontaneous differentiation - control	DMSO	MALDI TOF	S1b, 6, 7d	1
		MALDI FTICR	4	1
Spontaneous differentiation – PI-3 kinase inhibition	35 uM LY294002 in DMSO	MALDI TOF	S1, 6d, 7	1
Spontaneous differentiation – PI-3 kinase inhibition	100 uM LY294002 in DMSO	MALDI TOF	S1b, 5, 6, 7	1
Spontaneous differentiation – PEMT inhibition	50 uM 3-deazaadenosine	MALDI FTICR	4	1

Table S2. Lipid annotations for 16 selected features found in all 3 spontaneous differentiation experiments. The table shows MALDI FTICR *m/z* values for the species of interest, proposed annotation, main adduct type detected, experimental monoisotopic *m/z* value, elemental formula, mass error (ppm), annotation confidence level, MS/MS collision energies, and fragment ions used to determine lipid annotation. The confidence level for lipid annotation was assigned as (1) exact mass, isotopic pattern, and MS/MS spectrum of a chemical standard matched to the feature. (2) exact mass, isotopic pattern, retention time, and MS/MS spectrum matched to an in-house spectral database or literature spectra (3) putative ID assignment based only on elemental formula match. (4) unknown compound. Asterisks (*) designate compounds for which fatty acid annotation was obstructed by contamination with fragments from a co-selected precursor ion. No matches for the head group of *m/z* 940.5678 species were found in METASPACE and LIPID MAPS databases.

Precursor m/z (FTICR)	Proposed Annotation	Adduct Type	Monoisotopic Mass	Ion Mass	Elemental Formula	Mass Error (ppm)	Confidence Level	Fragment Ions Used to Determine Annotation (m/z)	Collision Energy (eV)
701.5122	PA (18:1)/(18:0) and PA (20:1)/(16:0)	$[M-H]^-$	702.52	701.5127	$C_{39}H_{75}O_8P$	-0.68	2	255, 281, 283, 309, 419, 437	15
722.5129	PE (P-16:0)/(20:4)	$[M-H]^-$	723.5203	722.513	$C_{41}H_{74}NO_7P$	-0.16	2	196, 259, 303, 419, 436	15
742.5385	PE (18:1)/(18:1)	$[M-H]^-$	743.5465	742.5392	$C_{41}H_{78}NO_8P$	-0.98	2	281	15
748.5281	PE O-38:6*	$[M-H]^-$	749.5359	748.5287	$C_{43}H_{76}NO_7P$	-0.76	3	N/A	15
778.5754	PE (22:4)/(P-18:0)	$[M-H]^-$	779.5829	778.5756	$C_{45}H_{82}NO_7P$	-0.28	2	155, 331	20
819.5177	PG (20:3)/(20:4)	$[M-H]^-$	820.5254	819.5182	$C_{46}H_{77}O_{10}P$	-0.56	2	227, 303, 305	20
821.5331	PG (18:1)/(22:5)	$[M-H]^-$	822.5411	821.5338	$C_{46}H_{79}O_{10}P$	-0.87	2	227, 281, 329	20
833.5181	PI (18:1)/(16:1)	$[M-H]^-$	834.5258	833.5186	$C_{43}H_{79}O_{13}P$	-0.55	2	223, 241, 253, 281	25
835.5346	PI (16:0)/(18:1)	$[M-H]^-$	836.5415	835.5342	$C_{43}H_{81}O_{13}P$	0.47	2	223, 241, 255, 281	25
859.5342	PI (16:0)/(20:3)	$[M-H]^-$	860.5415	859.5342	$C_{45}H_{81}O_{13}P$	0	2	223, 241, 255, 305	20
861.5499	PI (18:1)/(18:1)	$[M-H]^-$	862.5571	861.5499	$C_{45}H_{83}O_{13}P$	0.05	2	223, 241, 281	25
863.5649	PI (18:1)/(18:0)	$[M-H]^-$	864.5728	863.5655	$C_{45}H_{85}O_{13}P$	-0.7	2	223, 241, 281, 283	25
883.5336	PI (18:1)/(20:4)	$[M-H]^-$	884.5415	883.5342	$C_{47}H_{81}O_{13}P$	-0.68	2	223, 241, 281, 303	25
885.5494	PI (18:0/20:4)	$[M-H]^-$	886.5571	885.5499	$C_{47}H_{83}O_{13}P$	-0.51	2	223, 241, 283, 303	25
911.5661	PI 40:5*	$[M-H]^-$	912.5728	911.5655	$C_{49}H_{85}O_{13}P$	0.65	3	N/A	20
940.5678	Unknown	$[M-H]^-$	N/A	N/A	N/A	N/A	4	N/A	35

Table S3. Peak list of all molecular features detected in this study.

m/z	KM [CH2]	[Mass Defect]	KMD [CH2]	m/z	KM [CH2]	[Mass Defect]	KMD [CH2]
500.27813	499.71951	0.27813	0.28049	786.52896	785.65072	0.47104	0.34928
509.28829	508.71961	0.28829	0.28039	788.54462	787.66412	0.45538	0.33588
533.28824	532.69276	0.28824	0.30724	819.51770	818.55105	0.46608	0.44895
579.29371	578.64687	0.29371	0.35313	821.53310	820.49205	0.40925	0.42205
581.30937	580.66027	0.30937	0.33973	833.51810	832.58756	0.48172	0.41244
582.31263	581.66241	0.31263	0.33759	835.53460	834.60114	0.46589	0.39886
597.30429	596.63734	0.30429	0.36266	859.53420	858.57430	0.46594	0.42587
599.31994	598.65073	0.31994	0.34927	861.54988	860.58786	0.45012	0.41214
600.32333	599.65300	0.32333	0.34700	863.56490	860.58778	0.45021	0.41222
603.29369	602.62004	0.29369	0.37996	883.53360	882.54765	0.46579	0.45235
616.47092	615.78256	0.47092	0.21744	885.54986	884.56104	0.45014	0.43896
617.47426	616.78479	0.47426	0.21521	885.54986	885.56332	0.44675	0.43668
642.48654	641.76913	0.48654	0.23087	886.55325	885.56332	0.44675	0.43668
645.44982	644.72910	0.44982	0.27090	887.56546	886.57440	0.43454	0.42560
647.46552	646.74256	0.46552	0.25744	888.56904	887.57686	0.43096	0.42314
671.46552	670.71575	0.46552	0.28425	889.57271	888.57941	0.42729	0.42059
673.48117	672.72915	0.48117	0.27085	889.58128	888.58797	0.41872	0.41203
674.48454	673.73140	0.48454	0.26860	901.54488	900.53820	0.45512	0.46180
697.35681	696.57814	0.35681	0.42186	911.56610	910.54760	0.43453	0.45240
699.49687	698.71580	0.49687	0.28420	913.58135	912.56124	0.41865	0.43876
700.50023	699.71804	0.49977	0.28196	915.59697	914.57460	0.40303	0.42540
701.51251	700.72920	0.48749	0.27080	916.60035	915.57686	0.39965	0.42314
702.51588	701.73145	0.48412	0.26855	917.53982	916.51529	0.46018	0.48471
713.35178	712.55525	0.35178	0.44475	933.53481	932.49242	0.46519	-0.49242
716.52344	715.72337	0.47656	0.27663	934.53822	933.44947	0.46178	-0.49470
722.51290	721.70614	0.48709	0.29386	935.55043	934.50578	0.44957	0.49422
725.51259	724.70247	0.48741	0.29753	936.55389	935.50813	0.44611	0.49187
742.53909	741.70997	0.46091	0.29003	940.56752	939.51728	0.43248	0.48272
744.55476	743.72339	0.44524	0.27661	949.52971	948.46945	0.47029	-0.46945
748.52810	747.68559	0.47861	0.31441	951.54546	950.48295	0.45454	-0.48295
750.54421	749.70614	0.45579	0.29386	965.52474	964.44662	0.47526	-0.44662
752.55984	751.71952	0.44016	0.28048	967.53236	966.45201	0.46764	-0.45201
760.51330	759.66410	0.48670	0.33590	981.51943	980.42346	0.48057	-0.42346
766.53910	765.68317	0.46090	0.31683	1024.66141	1023.51727	0.32281	0.49601
768.55476	767.69659	0.44524	0.30341	1050.67718	1049.50399	0.38097	-0.44255
778.51438	777.64509	0.48562	0.35491	1051.68050	1050.50619	0.37710	-0.44530
